# A conserved antioxidant defense at the endoplasmic reticulum membrane

**DOI:** 10.1016/j.celrep.2026.117489

**Published:** 2026-06-04

**Authors:** Zhijian Ji, Henry de Belly, Taruna Pandey, Bingying Wang, Yao Tang, Jingxuan Yao, Shiya Xu, Kathy Li, Yanchang Bian, Shouhong Guang, Zhiyong Lou, Al Burlingame, Orion D. Weiner, Thomas D. Goddard, Dengke K. Ma

**Affiliations:** 1 Cardiovascular Research Institute, University of California San Francisco, San Francisco, CA, USA; 2 Department of Biochemistry and Biophysics, University of California San Francisco, San Francisco, CA, USA; 3 School of Life Sciences, Division of Life Sciences and Medicine, University of Science and Technology of China, Hefei, Anhui, China; 4 MOE Key Laboratory of Protein Science, School of Medicine, Tsinghua University, Beijing, China; 5 Department of Pharmaceutical Chemistry, University of California San Francisco, San Francisco, CA, USA; 6 Department of Physiology, University of California San Francisco, San Francisco, CA, USA; 7 Innovative Genomics Institute, Berkeley, CA, USA; 8 Lead contact

## Abstract

Oxidative protein folding in the endoplasmic reticulum (ER) is essential for eukaryotic cells yet generates hydrogen peroxide (H_2_O_2_), a reactive oxygen species. The ER-transmembrane protein that supports ER proteostasis and guards the cytosol for antioxidant defense remains unidentified. Here, we combine AlphaFold2 and functional screens in *C. elegans* to discover a previously uncharacterized and evolutionarily conserved protein ERGU-1 that fulfills these roles. Deleting ERGU-1 upregulates H_2_O_2_ and NRF2/SKN-1-dependent gene expression. ERGU-1 deficiency also impairs organismal reproduction and behavioral responses to H_2_O_2_. Both *C. elegans* ERGU-1 and human homolog TMEM161B localize to ER membranes, forming reticular networks. Human and *Drosophila* homologs of ERGU-1 rescue *C. elegans* mutant phenotypes, demonstrating ancient and conserved functions. In addition, purified ERGU-1 and TMEM161B exhibit redox-modulated oligomeric states. Together, our results reveal an ER-membrane-specific machinery, suggesting a conserved mechanism for maintaining ER redox homeostasis and proteostasis in animal cells.

## In brief

Ji et al. identify ERGU-1/TMEM161B as an evolutionarily conserved ER-transmembrane protein that modulates oxidative stress generated during oxidative protein folding. This critical guardian maintains cellular redox homeostasis, proteostasis, and optimal organismal reproduction across animal species.

## Graphical Abstract

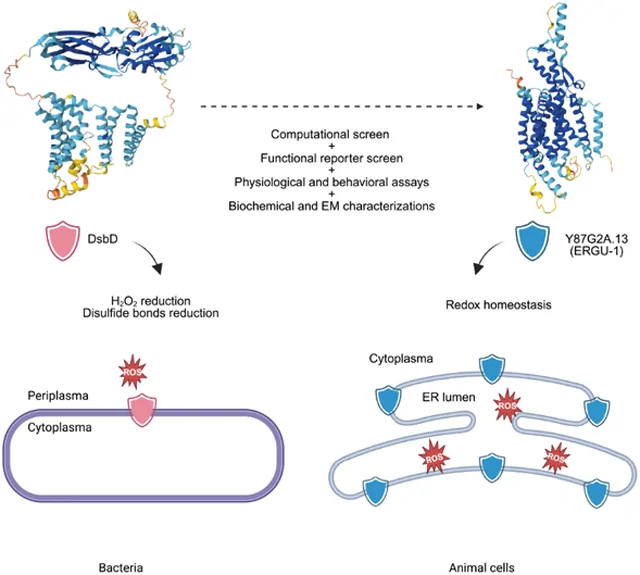

## INTRODUCTION

In eukaryotic cells, the endoplasmic reticulum (ER) plays a critical role in protein folding, a process essential for cell function but one that generates the reactive oxygen species hydrogen peroxide (H_2_O_2_).^1–3^ The ER-residing oxidase Ero1α stoichiometrically produces one molecule of H_2_O_2_ per disulfide bond introduced to nascent secreted or membrane proteins. Accordingly, the H_2_O_2_ concentration in the ER lumen is estimated to be approximately 700 nM, whereas cytosolic H_2_O_2_ concentration is estimated to be 2.2 nM at steady state.^4–6^ Cytosolic H_2_O_2_ levels are kept low through the concerted action of several buffering systems, notably peroxiredoxins and catalases. Catalase families are known to localize to peroxisomes, the cytosol, and mitochondria, but not to the ER.^7–10^ In *C. elegans*, no identified peroxiredoxin resides within the ER, suggesting that a distinct mechanism exists to protect the ER milieu from locally generated H_2_O_2_. While H_2_O_2_ can cross membranes by slow diffusion or aquaporin-facilitated transport,^11^ it remains unknown how ER membranes are guarded against the exceptionally high levels of ER H_2_O_2_, whose derivatives (e.g., hydroxyl radicals via Fenton reactions) can chemically attack and damage membrane lipids, nucleic acids, and proteins.

In bacteria, a family of transmembrane proteins (DsbD/ScsB)^12^ provides reducing equivalents for correct disulfide bond formation and peroxide reduction in the periplasm, which is considered the bacterial equivalent of eukaryotic ER. DsbD and ScsB carry multi-transmembrane segments and employ a series of cysteine pairs with differential redox potentials to relay electron transfer from cytosol to periplasm.^13^ Mammalian counterparts of DsbD and ScsB are proposed to exist yet remain unidentified.^14^ Our study addresses this gap in knowledge by identifying a previously uncharacterized and evolutionarily conserved *C. elegans* protein *Y87G2A.13* (named ERGU-1) as a key ER membrane-localized protein essential for redox homeostasis at ER and organismal functions. Furthermore, we reveal the structural and functional conservation of ERGU-1 homologs, including human TMEM161B, across various animal species, indicating an ancient role for ERGU-1 in maintaining cellular redox homeostasis.

## RESULTS

### Computational and genetic discovery of ERGU-1

We first performed a BLASTP search to identify potential eukaryotic sequence homologs of DsbD and ScsB. Such amino acid sequence similarity-based queries yielded no apparent broadly conserved eukaryotic homologs, even after adjusting sensitivity and specificity of the search (Figure S1). We reasoned that DsbD/ScsB and potential eukaryotic counterparts might not share primary protein sequence similarities owing to distant evolutionary divergence. Nevertheless, eukaryotic counterparts of DsbD/ScsB could exhibit structural and functional, rather than protein sequence features, common to DsbD/ScsB and known families of transmembrane oxidoreductases. These features include multi-transmembrane segments to facilitate intramembrane electron transfer, closely spaced cysteine clusters in different transmembrane segments in close proximity (5–10 Å), and broad evolutionary conservation in eukaryotic or multicellular organisms. Based on these features, we sought to leverage the availability of predicted protein structures by AlphaFold2 for nearly the entire *C. elegans* proteome^15^ and filter for DsbD/ScsB-like candidates for subsequent functional screens and validation in *C. elegans*.

To identify DsbD/ScsB-like candidates, we conducted a computational search of the *C. elegans* proteome based on the functional features common for DsbD/ScsB-like transmembrane oxidoreductases. Using the *C. elegans* UniProt reference proteome as a starting point (version 26, 19,827 proteins), we found all genes with at least four annotated transmembrane helices (3,177 proteins) and further filtered to those with at least two transmembrane helices, each containing at least two cysteines separated by at most two intervening residues (sequence patterns CC, CXC, or CXXC). For the 190 proteins meeting these criteria, we examined AlphaFold2 database-predicted structures and selected those in which cysteine pairs in two helices were no more than 10 Angstroms apart (SG to SG atom distance), producing 53 candidate genes (Figures 1A and S1). We implemented the computational screen using customized Python scripts (supplemental data file, Data S1) in UCSF ChimeraX.^16^

Among the 53 candidates with AlphaFold2-predicted structures passing the above screening criteria, we focused on the non-GPCR category with 11 hits for functional validation in *C. elegans* (Figures 1A and 1B). We performed RNA interference (RNAi) against genes encoding these 11 hits and tested if any can activate the transcriptional *gst-4p::GFP* reporter, which has been previously used to monitor excess oxidative stress and elevated levels of peroxide in *C. elegans*.^17^ We found that RNAi against one gene *Y87G2A.13* strongly activated *gst-4p::GFP* under standard laboratory conditions without exogenous oxidative stress, while RNAi against other genes or empty vector control did not (Figure 1B). Based on the T777T vector backbone with improved RNAi specificity and efficacy,^18^ we designed two independent RNAi constructs targeting different coding regions of *Y87G2A.13* and obtained similar results (Figures 1C and 1D). In addition, we used CRISPR-mediated genetic deletion to generate a null allele of *Y87G2A.13* and observed similar constitutive *gst-4p::GFP* activation in *Y87G2A.13* null mutants (Figures 1C and 1E). We also observed similar *gst-4p::GFP* activation by a protein-truncating mutation generated by the Million Mutation Project^19^ (Figures 1C, S2A, and S2B). The transcription factor SKN-1 is the *C. elegans* ortholog of NRF2, master regulator of antioxidant responses in mammals, and mediates *gst-4p::GFP* activation upon a variety of oxidative stresses.^20–22^ We found that RNAi against *skn-1* abolished the constitutive *gst-4p::GFP* activation in *Y87G2A.13* null and protein-truncating mutants (Figures 1F and S2C). N-acetyl-cysteine (NAC), a commonly used precursor for glutathione-based antioxidants and ROS/H_2_O_2_ scavenger in *C. elegans*,^23–25^ also strongly suppressed *gst-4p::GFP* activation in *Y87G2A.13* null mutants (Figure 1G). Taken together, these results show that reduced expression by RNAi or genetic deficiency of *Y87G2A.13*, one of the computationally identified ERGU candidates, constitutively activates the oxidative stress response in a manner that involves excess oxidants and activation of SKN-1. We hereafter refer to the Y87G2A.13 protein as ERGU-1 (ER guardian of redox homeostasis), given its redox-response mutant phenotype and additional lines of evidence below.

### Molecular and organismal roles of ERGU-1 in antioxidant defense in *C. elegans*

As intramembrane oxidoreductases, DsbD and ScsB transfer electrons via cysteine pairs as redox couples to reduce excess protein disulfide bonds and peroxides in bacterial periplasm.^12^ We thus assessed similar roles of ERGU-1 in ER protein folding stress and H_2_O_2_ balance, using the *hsp-4p::GFP* transcriptional reporter and a specific fluorescent sensor roGFP2-Orp1 for H_2_O_2_, respectively.^17,26^ We found that *ergu-1* genetic deletion or RNAi caused constitutive activation of *hsp-4p::GFP* in the absence of exogenous ER or protein folding stresses (Figures 2A and S3A). Using *C. elegans* strains expressing roGFP2-Orp1 to specifically monitor cytosolic levels of H_2_O_2_ through ratiometric dual-color fluorescence (Figure 2B), we found that *ergu-1* genetic deletion or RNAi led to markedly higher sensor oxidation-to-reduction ratios, indicating elevated H_2_O_2_ levels. An independent assay based on non-fluorescent compound dichlorofluorescein (DCFH) that reacts with H_2_O_2_ to produce fluorescent 2′,7′-dichlorofluorescein (DCF) showed similarly higher peroxide stress levels in *ergu-1* mutants (Figure 2C). Furthermore, we assayed effects of *ergu-1* loss (null, protein-truncating mutants or RNAi) on a comprehensive panel of stress-responding reporters, cell type-specific markers, and organelle membrane lipid sensors,^27–29^ but did not observe obvious phenotypic changes except in oxidative stress-related reporters (*gst-4p*:GFP, *hsp-4p*:GFP, roGFP2-Orp1, and Grx1-roGFP2) (Figures S2D, S2E, and S3A–S3M). Interestingly, though *ergu-1* deficiency strongly activated the SKN-1 target *gst-4p::GFP*, it failed to induce the NHR-49-dependent oxidative stress reporter *fmo-2p::GFP* (Figure S2G). Thus, the H_2_O_2_ generated by the loss of ERGU-1 likely engages a specific ER stress response rather than general oxidative stress response. These results suggest that ERGU-1 normally defends against specific thiol- or peroxide-related oxidative stress, consistent with its structural features and functional reporter regulation observed.

The AlphaFold2-predicted structure of ERGU-1 revealed two clusters of cysteine pairs C108/110 and C371/372 in its two transmembrane segments (Figures 1B and 3D). To evaluate the functional importance of such cysteine pairs for the antioxidant function of ERGU-1, we obtained CRISPR-mediated knock-in alleles converting cysteine to alanine at each site and crossed such cysteine mutants separately with reporter strains of *gst-4p::GFP*. We found that these cysteine mutants caused elevated levels of *gst-4p::GFP* (Figures 2E and 2F). Furthermore, while the *ergu-1* null mutant phenotype could be rescued by extrachromosomal expression of wild-type (WT) *ergu-1*, the cysteine mutant transgenes failed to rescue (Figure S4A), highlighting the functional necessity of these cysteine pairs. Although these cysteine residues are only partially conserved in the *ergu-1* orthologs across eukaryotic species (Figure S4D), the corresponding cysteine pairs are fully conserved in several closely related animal species (Figure S4E). These results suggest that the clusters of cysteine pairs of ERGU-1 functionally contribute to defending against oxidative stress, supporting their postulated functions in relaying electron transfer across ER membranes to reduce peroxide or disulfide stress from ER.

We next determined organismal roles of ERGU-1. Morphologically, the null and cysteine knock-in *ergu-1* mutants appear grossly normal. Previous studies have implicated roles of SKN-1 activation in several long-lived genetic mutants or by oxidative stress-induced hormesis in promoting longevity.^20^ We found that the *ergu-1* null mutants, with control or *skn-1* RNAi, exhibited largely normal lifespans (Figure S3H) under standard laboratory conditions at 20°C fed with OP50 on FuDR-NGM plates. However, *ergu-1* mutants showed heightened sensitivity when other redox regulators were compromised: *ergu-1* mutants exhibited increased *gst-4p::*GFP induction compared to WT upon RNAi knockdown of various ER and cytosolic oxidant-buffering genes (*ero-1, gcs-1, gsr-1, gpx* family, and *pdi* family) (Figure 2G). Moreover, *ergu-1* mutants subjected to *gpx-1* RNAi displayed a much stronger *gst-4p::*GFP induction than RNAi-treated WT animals, indicating that ERGU-1 acts in concert with GPX-1 to overcome ER-derived oxidative stress (Figure 2G). Notably, assay quantification of reproductive capacity revealed a markedly reduced brood size of *ergu-1* null mutants. Consistently, the *ergu-1* cysteine mutants (C108A/C110A and C371A/C372A) exhibited similar reduction in brood sizes (Figure 2H). This reduced brood size phenotype could be partially alleviated by 10 mM antioxidant NAC treatment (Figure 2H). By contrast, treatment with exogenous oxidative stressor paraquat decreased brood size more severely in *ergu-1* mutants than WT (Figure S3I). In addition, we measured the locomotion behavior in young adult hermaphrodites upon acute exposure to exogenous H_2_O_2._ The *ergu-1* null mutants exhibited a markedly blunted slowing of movement in response to H_2_O_2_, without apparently altered effects on neuronal development, cytoskeletal structure, or baseline neuronal activity (Figures S3J–S3N). These results indicate that ERGU-1 is critical for maintaining normal specific organismal functions in reproduction and behavioral responses to H_2_O_2_.

### Subcellular localization of *C. elegans* ERGU-1 at the ER membrane

To elucidate the tissue distribution and subcellular localization of ERGU-1, we constructed both a transcriptional (*ergu-1p::GFP*, fusing the *ergu-1* promoter to the green fluorescent protein GFP) and a translational reporter (*ergu-1p::ergu-1::GFP*, fusing GFP to the C terminus of ERGU-1 under the control of its native promoter) (Figure 3A). We microinjected the constructs and integrated the transgenic extrachromosomal arrays to the genome at low copy number to ensure the faithful recapitulation of ERGU-1’s endogenous expression pattern. Both the transcriptional and translational reporters similarly revealed *ergu-1* expression in major metabolic tissues, including the intestine, body wall muscles, and the spermatheca (Figures 3B and 3C). As expected, RNAi against *ergu-1* abolished GFP signals from the *ergu-1*p:*ergu-1*:GFP transgene (Figure S3B). Notably, the tissues expressing *ergu-1* including body wall muscles, intestine, spermatheca, and excretory cells generate elevated levels of H_2_O_2_ during protein folding, as indicated by roGFP2-Orp1 (Figure 2B).

To ascertain ERGU-1’s subcellular localization, we crossed the translational reporter with a previously well-characterized ER membrane marker SEL-1(1–79)::mCherry::HDEL.^29,30^ High-resolution dual-fluorescence confocal microscopy and line-scanning analysis revealed marked co-localization of *ergu-1*p:*ergu-1*:GFP and SEL-1(1–79)::mCherry::HDEL in a net structure-like reticulum pattern (Figures 3D–3I and S5A). ERGU-1::GFP showed particularly prominent intensities in anterior and posterior intestinal cells, consistent with effects we observed with roGFP2-Orp1 (Figure 2B). We confirmed the ER membrane-specific localization of ERGU-1 by imaging ERGU-1::GFP alongside RFP markers specific for other organelles, including the mitochondria, lysosomes, and plasma membrane (Figure S5B). The ER membrane localization aligns markedly well with ERGU-1’s proposed functional role in mitigating the detrimental effects of H_2_O_2_ produced during protein folding within the ER lumen. To address potential ER membrane-localized changes in oxidative stresses, we generated ER-proximal redox sensor strain GRX1::roGFP::SPSC-1. We observed markedly higher oxidation-to-reduction ratio of roGFP upon *ergu-1* and *nduf-7* (positive control) RNAi treatment (Figures 3J and 3K). Furthermore, biochemical purification of *ergu-1*p:*ergu-1*:GFP using GFP-trap affinity chromatography revealed a redox-sensitive oligomeric organization of ERGU-1 (Figure 4F), with monomers being prevalent under reducing conditions. Together, the ER membrane-specific localization, redox regulation, and antioxidant function support ERGU-1 as a guardian of redox homeostasis.

### Evolutionarily conserved features of ERGU-1 family proteins

We next investigated the evolutionary conservation of ERGU-1. First, we constructed a multiple sequence alignment and a maximum likelihood-based phylogenetic tree encompassing ERGU-1 and its homologs from diverse animal species (Figures S4D; Figure 4A). This analysis revealed a high degree of sequence conservation across the ERGU-1 protein family, suggesting a potentially ancient and functionally important role. For instance, the mouse ERGU-1 homolog Tmem161b and human TMEM161B exhibit 93.6% amino acid percent identities, while *C. elegans* ERGU-1 and human TMEM161B exhibit 27.9% amino acid percent identities. To experimentally test evolutionary conservation of the ERGU-1 protein family, we functionally complemented the *C. elegans ergu-1* mutant phenotype with homologs from other species. We expressed the *ergu-1* homologs *emei* and *TMEM161B* from *Drosophila* and humans, respectively, under the control of endogenous *C. elegans ergu-1* promoter, in the *ergu-1* mutant background carrying the *gst-4p::GFP* reporter (Figure 4B). We found that transgenic expression of these homologs rescued the constitutively activated *gst-4p::GFP* phenotypes, indicating normalized oxidative stress in *C. elegans ergu-1* mutants (Figure 4C). This successful cross-species complementation supports the functional conservation of ERGU-1 and its homologs in multicellular organisms.

To further explore the conservation of ERGU-1 function, we focused on human TMEM161B, which appears more closely related to ERGU-1 than its paralog TMEM161A (Figure 4A). To mirror our *C. elegans* studies, we first examined TMEM161B’s subcellular localization in mammalian cells. We expressed mCherry-tagged TMEM161B cDNA under the control of a CMV promoter in HEK293T and U2OS human osteosarcoma cells. Immunofluorescence imaging revealed a striking colocalization with established ER-GFP markers (Figures 4D and S6F). Furthermore, to assess the potential antioxidant function of TMEM161B analogous to *C. elegans* ERGU-1, we employed V5-tagged TMEM161B cDNA expressed in HEK293 cells and monitored cytosolic H_2_O_2_ using the roGFP2-Orp1 sensor. Expression of human TMEM161B led to a strong suppression of H_2_O_2_-induced roGFP2 oxidation (Figures 4E and S6G). We also used the AlphaFold3 webserver^31^ to model various ligand interactions with TMEM161B and ERGU-1 and identified heme-binding pockets coordinated by a highly conserved tyrosine residue (Y454) (Figures S4B–S4D). Functionally, the Y454A mutant ERGU-1 could not rescue the *ergu-1* mutant phenotype on *gst-4p::GFP* activation (Figure S4C). Beyond the redox-sensitive oligomeric organization of ERGU-1 (Figure 4F), purified human TMEM161B exhibited native oligomerization under normal conditions and dissociation into monomers in a reducing environment, as demonstrated by size exclusion chromatography (Figure 4G). Interestingly, cryo-electron microscopy analysis revealed a filamentary oligomeric form of TMEM161B under non-reducing conditions, while treatment with DTT-induced monomerization (Figure 4H). These findings indicate that human TMEM161B recapitulates several key features of *C. elegans* ERGU-1, including ER membrane localization, antioxidant functions, and redox-modulated oligomeric states, providing further evidence for the evolutionary conservation of ERGU-1/TMEM161B structure and functions in antioxidant defense across the animal kingdom.

Mechanistically, we gained additional insights into how ERGU-1/TMEM161B may coordinate with their physical interactors. Using ERGU-1::GFP as a bait, we performed co-immunoprecipitation coupled with mass spectrometry and identified metabolic enzymes such as glutamate dehydrogenase (GLUD) and glyceraldehyde-3-phosphate dehydrogenase (GAPDH) as ERGU-1-associated proteins (Table S1). These enzymes promote the generation of NADPH and NADH, respectively, suggesting that ERGU-1 may harness reducing equivalents directly from cytosolic metabolism. A recently published human protein-protein interaction dataset^32^ predicts with high-confidence scores from independent models that TMEM161B interacts with TMX2, an ER-resident thioredoxin-like protein (Figures S7A and S7B). To test whether this relationship is possibly conserved in *C. elegans*, we performed RNAi knockdown of *ergu-1* and *C35D10.10* (the worm ortholog of TMX2) in *hsp-4p::GFP* animals. We found that both knockdowns induced *hsp-4p::GFP* expression to a similar extent (Figure S7C), supporting the idea that ERGU-1 and TMX2 may act in the same pathway to maintain ER homeostasis. Together, these results support a model in which ERGU-1 functions analogously to bacterial DsbD: ERGU-1 may drive reduction of ER-derived peroxide and thioredoxin-like proteins (such as C35D10.10/TMX2), thereby enhancing the ER’s antioxidant capacity. This mechanism would operate in parallel with the classical cytosolic thioredoxin and glutathione systems, underscoring the redundancy and robustness of ER redox homeostasis.

## DISCUSSION

In this study, we identify ERGU-1, a previously uncharacterized and evolutionarily conserved protein, as a critical component of the ER-resident system for redox homeostasis and proteostasis in animal cells. We used a combination of computational and functional screening to pinpoint ERGU-1 among the entire UniProt-defined *C. elegans* proteome. Determining the initial list of ERGU candidates for functional validation exemplifies the utility of AlphaFold2 in guiding protein function discovery. Despite known limitations in accuracy compared to experimentally determined structures, AlphaFold2 predictions can guide researchers toward promising directions for functional studies, advancing our understanding of fundamental biological processes and potentially enabling new therapeutic strategies.^31,33–35^ We designed our filtering criteria for ERGU candidates based on empirical knowledge gained from DsbD/ScsB and membrane oxidoreductases. A similar strategy with modified criteria could be applied to discover other protein functions beyond ERGU-1.

Despite the known generation of H_2_O_2_ during oxidative protein folding, the identity of the ER membrane-resident protein responsible for managing this stress has remained elusive. Our work bridges this gap by identifying ERGU-1 as such a key player, which harbors functional and structural features characteristic of transmembrane oxidoreductases. Importantly, genetic deletion of ERGU-1 led to constitutive activation of oxidative stress- and ER stress-response reporters, elevated cytosolic H_2_O_2_ levels, and organismal phenotypes in reproduction and behaviors. These findings strongly suggest ERGU-1’s crucial role in maintaining ER redox homeostasis and proteostasis, potentially through electron transfer via cysteine pairs across ER membranes to manage luminal oxidative stress. As loss of ERGU-1 also activated the *hsp-4p::GFP* reporter indicative of unfolded protein stress, it is plausible that ERGU-1 provides reducing equivalents from cytosolic electron donors for additional ER substrates other than H_2_O_2_, e.g., misfolded proteins, GSSG or certain peroxide by-products that ScsB or DscB are known to reduce.^12,36–38^ However, neither ScsB nor DsbD is known to bind to heme, unlike the *C. elegans* and human homologs of ERGU-1. We thus postulate that eukaryotic ERGU-1/TMEM161 homologs may have evolved additional heme-binding capacity that likely facilitates heme and redox homeostasis or directly.

The subcellular localization of ERGU-1/TMEM161 at the ER membrane is well suited for its antioxidant function. By residing at the site close to H_2_O_2_ generation, ERGU-1 may efficiently manage it, safeguarding the ER membrane and nearby cytosol from oxidative damage. Furthermore, the redox-sensitive nature of the tetrameric ERGU-1 organization suggests a potential regulatory mechanism for ERGU-1 function. Under more oxidizing conditions, the tetrameric form appears to be more prevalent, potentially enhancing ERGU-1’s activity to safeguard redox homeostasis against excess peroxide stress. Interestingly, our findings on ERGU-1/TMEM161B and its proximity to H_2_O_2_ generation in the ER align with recent studies suggesting a limited role for mitochondrial ROS in causing global oxidative stress and nuclear DNA damage.^39^ This highlights the ER as a major source of cellular oxidative stress impacting nuclear membranes and the genome. By effectively mitigating ER-derived H_2_O_2_, ERGU-1 may play a critical role in shielding the genome from oxidative damage. Furthermore, ribosomal RNA, densely packed on ER membranes and crucial for protein synthesis, is another likely target of such oxidative damage. It remains to be determined whether ERGU-1 protects against oxidative damage in general or more specifically biases toward certain biomolecules. In specific tissues (e.g., intestine), excess peroxide stress caused by loss of ERGU-1 may also activate multiple compensatory antioxidant pathways, including SKN-1/NRF2, and recruit additional mechanisms to safeguard cellular and organismal functions.

The high degree of sequence conservation observed within the ERGU-1 protein family across diverse animal species underscores its potential evolutionary ancestry and functional importance. The successful complementation of the *C. elegans ergu-1* mutant phenotype with homologs from *Drosophila* and humans further strengthens this notion. Although we did not find any apparent orthologues of ERGU-1 in eukaryotic fungi, our findings indicate evolutionary conservation of ERGU-1/TMEM161 in metazoans, emphasizing its critical and previously undescribed role in maintaining cellular redox balance. Mutations in the *Drosophila* orthologue *emei* impair ER calcium dynamics,^40^ whereas mutations in its vertebrate orthologue *Tmem161B* cause severe pathological cardiac arrhythmias in mice and brain polymicrogyria in humans.^41–44^ Our studies suggest that these previously unexplained phenotypic defects might be mechanistically caused by defects in ERGU-1/TMEM161’s homologous functions in redox homeostasis.

### Limitations of the study

Although this study establishes ERGU-1/TMEM161B as a conserved ER-membrane antioxidant protein, several limitations remain. First, the AlphaFold-guided predictions require further validation by cryo-EM and biophysical characterization of protein structural properties. Second, the enzymatic mechanism and electron donor(s) of ERGU-1 have not yet been defined, and *in vitro* reconstitution will need to determine its precise biochemical targets and how it acts in coordination with its partners to maintain heme homeostasis or redox balance at ER. Third, while *C. elegans* and cell-based assays support its conserved role in redox regulation, the physiological relevance of TMEM161B in mammalian ER homeostasis and stress responses remains to be fully tested in vivo.

## RESOURCE AVAILABILITY

### Lead contact

Requests for further information and resources should be directed to and will be fulfilled by the lead contact, Dengke K. Ma (dengke.ma@ucsf.edu).

### Materials availability

All unique/stable reagents generated in this study are available from the lead contact with a completed materials transfer agreement.

### Data and code availability

- Mendeley Data: original western blot images and microscopy data have been deposited at Mendeley at https://data.mendeley.com/datasets/fgg6sft85z/1.
- Code: all original code has been deposited at Zenodo at https://doi.org/10.5281/zenodo.19673017 and is publicly available as of the date of publication.
- Any additional information required to reanalyze the data reported in this paper is available from the lead contact upon request

## STAR★METHODS

### EXPERIMENTAL MODEL AND STUDY PARTICIPANT DETAILS

#### C. elegans

*C. elegans* strains were maintained under standard laboratory conditions unless otherwise specified. The N2 Bristol strain was used as the reference wild-type. Feeding RNAi was performed as previously described.^45^ Transgenic strains were generated by germline transformation as described.^46^ Transgenic constructs were co-injected (at 5–10 ng/μL) with dominant *unc-54p*:mCherry or *myo-2p*:mCherry, and stable extrachromosomal lines of fluorescent animals were established for UV-mediated genome integration. Genotypes of strains used are as follows: *ergu-1(ust572, syb9153, syb9160), dvIs19 [gst-4p::GFP], zcIs4 [hsp-4::GFP], jrIs10 [unc119(*+*) rps-0p::roGFP2-Orp1], dmaIs160 [ergu-1p:: ergu-1::gfp; unc-54p::mCherry], dmaEx701[ergu-1p::hsTMEM161B::ergu-1utr; myo-2p::mCherry], dmaEx702 [ergu-1p::ergu-1::ergu-1utr; myo-2p::mCherry], dmaEx704 [ergu-1p::Dm-161:: ergu-1utr; myo-2p::mCherry]*. CRISPR/Cas9-mediated genome editing was performed. Knockout alleles were generated by deleting the coding sequence of *ergu-1* in Guang lab, and cysteine-to-alanine substitutions were introduced by homology-directed repair using synthetic oligonucleotide templates by SunyBiotech platform (Fuzhou, China). All mutations were confirmed by Sanger sequencing.

### METHOD DETAILS

#### AlphaFold-assisted computational screen

A comprehensive computational screen of the *Caenorhabditis elegans* proteome was performed to identify potential DsbD/ScsB-like proteins. The reference proteome utilized for this analysis was the *C. elegans* UniProt reference proteome (version 26), encompassing 19,827 genes. Initial filtering was conducted to select genes encoding proteins with a minimum of four annotated transmembrane helices, resulting in a subset of 3,177 genes. Subsequent filtering criteria focused on the presence of transmembrane helices containing cysteine residues, specifically targeting helices with at least two cysteines separated by no more than two intervening residues, corresponding to the sequence motifs CC, CXC, or CXXC. This filtering narrowed the candidate list to 190 genes. The structural analysis was conducted using predicted protein structures from the AlphaFold2 Database. For each of the 190 genes, the predicted structures were examined to determine the spatial proximity of cysteine pairs located within the transmembrane helices. Specifically, structures in which the sulfur atoms (SG) of cysteine pairs were within 10 angstroms of each other were identified. This criterion was met by 53 candidate genes. We noted that using different versions of UniProt may yield small variations in the number of hits, although the overall candidate list remains robust.

The computational search and structural analysis were executed using a custom Python script (Data S1, https://doi.org/10.5281/zenodo.19673017). The script reads the UniProt *C. elegans* reference proteome which includes all transmembrane helix annotations and sequences provided in a single XML file (version 2023_02, https://ftp.uniprot.org/pub/databases/uniprot/knowledgebase/reference_proteomes/Eukaryota/UP000001940/UP000001940_6239.xml.gz). Predicted structure models were obtained for the entire *C. elegans* proteome from the AlphaFold 2 Database bulk download site (version 4, https://ftp.ebi.ac.uk/pub/databases/alphafold/v4/UP000001940_6239_CAEEL_v4.tar). For each gene passing the transmembrane helix and sequence filtering steps the Python script (Data S1) executed within ChimeraX version 1.5 measured cysteine sulfur atom distances in the predicted structure models to further filter the results. Visualization and structural analyses of the resulting 53 candidate structures were performed interactively using UCSF ChimeraX software.

#### RNA interference (RNAi) cloning and screen

RNAi and screen for hits upregulating *gst-4p*:GFP were performed by feeding worms with *E. coli* strain HT115 (DE3) expressing double-strand RNA (dsRNA) targeting endogenous genes. Briefly, dsRNA–expressing bacteria were replicated from the Ahringer library to LB plates containing 100 μg/mL ampicillin at 37°C for 16 h. A single clone was picked to LB medium containing 100 μg/mL ampicillin at 37°C for 16 h and positive clones (verified by bacteria PCR with pL4440 forward and pL4440 reverse primers) were spread onto NGM plates containing 100 μg/mL ampicillin and 1 mM isopropyl β-D-1-thiogalactopyranoside for 24 h. Developmentally synchronized embryos from bleaching of gravid adult hermaphrodites were plated on RNAi plates and grown at 20°C. Worms were collected for imaging at indicated stages.

To make the *ergu-1* RNAi-1 and RNAi-2 plasmids, RNAi-1 targeting sequences are PCR-amplified with primers 5′- GGCCCCCCCTCGAGGTCGACAGGAGACGATGCTATAAACTATGCT -3′ and 5′- TCCACCGCGGTGGCGGCCGCCGTGACGTGTTTTCAGCTCG -3′ from N2 gDNA and subcloned into the SalI and NotI sites of a digested T777T vector with Promega T4 DNA ligase (Promega, M1801). RNAi-2 targeting sequences are PCR-amplified with primers 5′- GGCCCCCCCTCGAGGTCGACATGCCAAAATGAATGCGGGC -3′ and 5′- TCCACCGCGGTGGCGGCCGCACGTGGTGATATGCCTGGTG -3’.

#### Fluorescence microscopy and H_2_O_2_ sensor imaging

SPE confocal (Leica) and epifluorescence microscopes were used to capture fluorescence images. Animals were randomly picked at the same stage and treated with 1 mM levamisole in M9 solution, aligned on a 2% agar pad on a slide for imaging. Identical settings and conditions were used to compare experimental groups with control. For quantification of GFP fluorescence, animals were outlined and quantified by measuring gray values using the ImageJ software. The data were plotted and analyzed by using GraphPad Prism10. For *jrIs10 [unc119(*+*) rps-0p::roGFP2-Orp1]* strain, orp1-roGFP2 was excited sequentially at 405 and 488 nm and emission was recorded at 500–540 nm. Fifteen images were sequentially captured at 1-micrometer z-intervals and subsequently stacked to form a composite image.

#### Western blotting

For SDS-PAGE samples, stage-synchronized animals for control and experiment groups were picked (*n* = 50) in 60 μL M9 buffer and lysed directly by adding 20 μL of 4x Laemmli sample buffer (1610747, Bio-Rad). Protein extracts were denatured at 95°C for 10 min and separated on 10% SDS-PAGE gels (1610156, Bio-Rad) at 80 V for ~40 min followed by 110 V for ~70 min. The proteins were transferred to a nitrocellulose membrane (1620094, Bio-Rad) at 25 V for 45 min by Trans-Blot Turbo Transfer System (Bio-Rad). The NC membrane was initially blocked with 5% non-fat milk and 2% BSA (A4503, Sigma (v/v)) in tris-buffered saline with 0.1% Tween 20 (93773, Sigma) (TBST) at room temperature for 1 h. Proteins of interest were detected using antibodies against GFP (A6455, Invitrogen) or V5 (13202T, Cell Signaling Technology) in cold room for overnight. After three washes of 5 min each with tris-buffered saline with 0.1% Tween 20, anti-mouse IgG, HRP-linked Antibody (7076, Cell Signaling Technology) was added at a dilution of 1:5000. For DTT treatment, worm lysates were treated with 10 mM DTT and boiled at 95°C for 10 min.

#### Mammalian cell culture experiments

Human embryonic kidney (HEK) 293T cells and osteosarcoma U2OS cells were maintained in Dulbecco’s modified Eagle’s medium with 10% inactive fetal bovine serum and penicillin-streptomycin (Gibco, Grand Island, 15140122) at 37°C supplied with 5% CO2 in an incubator (Thermo Fisher Scientific) with a humidified atmosphere. Cells were washed once using PBS and digested with 0.05% trypsin EDTA (Gibco) at 37°C for routine passage of the cells. All cells were transfected with 3 μL LipoD293 (SignaGen, SL100668) per 1 μg DNA mixture. HEK293T cells were transfected by the pLX304-TMEM161A-V5 (DNASU, HsCD00441633) or pLX304-TMEM161B-V5 (DNASU, HsCD00444935) and collected 2 days after transfection. HEK293T and U2OS cells were transfected by the pHAGE2-TMEM161A-gfp/mCherry or pHAGE2-TMEM161B-gfp/mCherry and collected for imaging 2 days after transfection. pHAGE2-TMEM161A/B-GFP/mCherry was constructed by inserting TMEM161A or TMEM161B into pHAGE2-gfp/mCherry plasmids. TMEM161A was PCR-amplified with the primers 5′- TAATTAAACCTCTAGAGCCACCATGGCGGTCCTCGG -3′ and 5′- CTCACCATAGCTCGAGacccagacccGGAGCCTGCCAAGTGC -3′ from pLX304-TMEM161A-V5 mentioned before. TMEM161B was PCR-amplified with the primers 5′- TAATTAAACCTCTAGAGCCACCATGGGTGTGATAGGTATACAGC -3′ and 5′- CTCACCATAGCTCGAGacccagacccTGCCACAGTCAGATACTGGT -3′ from pLX304-TMEM161B-V5 described above. The ER marker used was mEmerald-ER-5 (Addgene 54083).

#### Quantification of brood size

The brood size assay was carried out according to the standard protocol. Briefly, single L4 worms of different strains were individually placed in 60 mm Petri plates and kept in the incubator at 20 C. Then, the worms were transferred to a new OP50 containing plate each day at the same time until day 6. Progeny were scored after 3 days and plotted for each day and the total sum of progenies. For NAC treatment the plates were supplemented with 10 mM of NAC during the entire duration of the assay. For H_2_O_2_ treatment the plates were supplemented with 10 mM of H_2_O_2_ during the assay. For paraquat assays, worms were exposed to 10 mM paraquat continuously throughout the brood size assay (days 1–6). Each experiment was repeated 3 times as independent biological replicates with more than 5 animals per group.

#### Behavioral assay

Synchronized young adult day 1 (D1) hermaphrodites were transferred to a fresh NGM plate seeded with a small OP50 bacterial lawn and allowed to settle for at least ten minutes to recover at room temperature. Moving average speed, and track length of *C. elegans* were monitored for 10 min and were analyzed using WormLab. For H_2_O_2_ treatment, worms were treated with 10 mM H_2_O_2_ for 30 min before assay.

#### Lifespan analysis

For lifespan assays, animals were cultured under non-starved conditions for at least 2 generations before life span assays. For normal NGM life span assay, stage-synchronized L4 stage animals (*n* ≥ 50) were picked to new NGM plates seeded with. OP50 containing 50 μM 5-fluoro-2′-deoxyuridine (FUDR) to prevent embryo growth at. 20°C incubator. Animals were scored for survival every 24 h. Animals failing to respond to repeated touch of a platinum wire were scored as dead. Three biological replicates were performed, with population sizes larger than 50 in each trial.

#### DCFDA ROS detection

ROS staining in live worms was carried out as in standard protocol with minor modifications. Briefly, day 1 worms were washed three times with M9 and then transferred into 200 μL of M9 buffer containing 10 mM H2DCFDA (carboxy-H2DCFDA [5-(and-6)-carboxy-2′,7′-dichlorodihydrofluorescein diacetate) in an 1.5 mL Eppendorf tube and incubated in the dark for 3 h. The worms were randomly selected and treated with 10 mM sodium azide (Sigma-Aldrich) in M9, symmetrically aligned on 2% agar pads on slides for imaging the oxidized dichlorofluorescin (DCF).

#### Expression and purification of TMEM161B for cryo-EM

The complementary DNA encoding human TMEM161B was cloned into a modified pDNA3.1 vector with a C-terminal twin-strep tag and an HA tag. For expression, Expi293F cells (Thermo Fisher Scientific) cultured in Freestyle293 Expression Medium were transfected with the vector DNA/polyethylenimine (1 μg DNA per mL culture, w/w = 1:3) complex at a cell density of ~1.0 × 106 cells per mL and incubated at 37°C under 8% CO2 with agitation at 100 r.p.m for 60 h. Cell pellets were resuspended in buffer A (25 mM HEPES pH 7.5, 150 mM NaCl, protease inhibitor cocktail) and were disrupted by sonication. For TMEM161B monomer purification, an extra incubation with 5 mM DTT at room temperature after sonication was required. The cell lysate was then spun at 150,000× g for 1h to sediment crude membranes. The membrane pellet was mechanically homogenized in buffer A. The suspension was solubilized in 1% (w/v) 1.0% DDM (Anatrance) and 0.1% CHS (Anatrance) for 60 min at 4°C. The solubilized material was centrifuged at 100,000× g for 30 min, and the supernatant was incubated with Strep-TactinXT resins (iba) for 2 h at 4°C. Resins were then washed with 20 column volumes of buffer B (25 mM HEPES pH 7.5, 0.025% DDM, 0.0025% CHS, 150 mM NaCl). The protein was eluted with buffer B supplied with 50 mM D-biotin, concentrated, and further purified by gel filtration chromatography on a Superdex 200 increase column equilibrated with wash buffer B. The peak fractions of protein were pooled and concentrated to ~5.0 mg/mL.

#### Cryo-EM sample preparation and observation

For each sample, a 3-μL aliquot was applied onto a glow-discharged Quantifoil grid (R1.2/R1.3 300 mesh, Au), blotted for 4.5–5.5 s in 100% humidity at 4°C, and plunged into liquid ethane using a Vitrobot MkIV (Thermo Fisher Scientific). Cryo-EM micrographs were obtained by using a Talos Arctica G2 microscope (Thermo Fisher Scientific) running at 200 kV and a 300 kV Titan Krios microscope (Thermo Fisher Scientific).

#### Phylogenetic and sequence analysis

To assess the evolutionary conservation of ERGU-1, we first identified homologs in representative animal species by BLASTP and reciprocal BLASTP searches using *C. elegans* Y87G2A.13 as a query. Full-length protein sequences were retrieved from UniProt or NCBI RefSeq databases. Multiple sequence alignment was performed using Clustal Omega (version 1.2.4) with default parameters. The alignment was visualized using Jalview, and colors were applied by conservation and clustering. A maximum-likelihood phylogenetic tree was generated from the aligned sequences using MEGA11 software, The tree includes a scale bar representing a branch length of 0.2, which corresponds to 20% sequence divergence between aligned proteins. The multiple sequence alignment underlying this analysis is provided in Figure S7 and in the link https://www.ebi.ac.uk/jdispatcher/msa/clustalo/summary?jobId=clustalo-I20251028-184456-0417-81877365-p1m.

### QUANTIFICATION AND STATISTICAL ANALYSIS

Data were analyzed using GraphPad Prism 10.0 Software (Graphpad, San Diego, CA) and presented as means ± SEM unless otherwise specified, with *p* values calculated by unpaired two-tailed t-tests (comparisons between two groups), one-way ANOVA (comparisons across more than two groups) and adjusted with Bonferroni’s corrections. Boxplots are presented as min to max, showing all points. Statistical details of experiments can be found in the figure legends.

## Supplementary Material

Document S1. Figures S1–S7 and Table S1 **Fig. S1. AlphaFold2-based computational screen for ERGU candidates.** (**A**) BLASTP search yielding no apparent broadly conserved eukaryotic homologs of DsbD/ScsB based on protein sequence similarities, prompting a search for DsbD/ScsB homologs based on functional and structural features using AlphaFold2-predicted structures. (**B**) Predicted structures of the 53 ERGU candidates identified. Transmembrane helices are shown in blue, cysteine residues in yellow, and the remainder of the protein in gold. (**C**) Table listing the annotated functions of the 11 non-GPCR ERGU candidates.
**Fig. S2. Phenotypes of ERGU-1 protein-truncating *ergu-1(gk840471)* mutants.** (**A**) Representative epifluorescence images showing *gst-4p::GFP* activation in *ergu-1*(*gk840471*) mutants (n=15 for each group). (**B**) Quantification of *gst-4p*::GFP fluorescence intensities in WT and *ergu-1*(*gk840471*) mutants indicated (n=15 for each group). (**C**) Representative epifluorescence images showing *gst-4p::*GFP decrease by *ero-1* RNAi and increase by *gcs-1* RNAi (**D**) Representative epifluorescence images showing *gst-4p::GFP* activation in *ergu-1*(*gk840471*) mutants requires *skn-1.* (**E**) Representative fluorescence and corresponding bright-field images showing *hrg-1p*::GFP reporter expression in control and ergu-1 RNAi-treated worms. (**F**) Representative confocal fluorescence images showing no apparent reporter (Golgi apparatus-marking Golgi::GFP, apical and endosomal membrane-marking *akt-ph*::GFP, yolk organelle-marking *vit-2*::GFP and autophagosome-marking *sqst-1*::mOrange2) alteration in *ergu-1*(*gk840471*) mutants. (**G**) Representative fluorescence and corresponding bright-field images showing *fmo-2p*::GFP reporter expression in control and ergu-1 RNAi-treated worms. *P* value was determined by an unpaired *t*-test, two-tailed (comparison between two groups). [(A), (B), (C), (E) and (G)] Scale bars, 100 μm. (D) Scale bars, 20 μm.
**Fig. S3. Phenotypic consequences of RNAi or mutant *ergu-1*.** (**A**) Representative epifluorescence images showing increased *hsp-4p::GFP* upon *ergu-1* RNAi treatment. (**B**) Representative fluorescence images of *ergu-1p*::*ergu-1::GFP* showing abolished GFP signals by *ergu-1* RNAi. (**C**) Representative fluorescence images of Vitamin B12 stress reporter *acdh-1p*:: *GFP* showing unaltered GFP signals by *ergu-1* RNAi. (**D** and **E**) Representative fluorescence images of glutathione stress GSSG/GSH reporters showing elevated oxidative stress in *ergu-1* null mutants (D) and quantification (n=10) (E). (**F**) Representative fluorescence images of *ergu-1p*::*ergu-1::GFP* showing reporter up-regulation by oxidative stressors (0.5% NaAsO_2_). (**G**) Lifespan curves of wild-type and *ergu-1* null mutants with control or *skn-1* RNAi showing comparable lifespans in wild-type and *ergu-1* null mutants and reduction by *skn-1* RNAi. (**H**) Lifespan curves of wild-type and *ergu-1* protein-truncating mutants (*gk840471*) with control or *skn-1* RNAi comparable lifespans in wild-type and *ergu-1(gk840471)* mutants and reduction by *skn-1* RNAi (n ⩾ 50). (**I**) Quantification of brood sizes in wild type, *ergu-1* null and cysteine mutants upon 10 mM paraquat treatment. *P* value was determined by one-way ANOVA (comparison between multiple groups). (**J**) Quantification of average moving speed in wild-type, ergu-1 null and above strain treated with 10 mM H2O2. (**K**) Representative fluorescence images of cytoskeletal actin *act*-*1*::GFP reporters showing no apparent alteration by *ergu-1* RNAi. (**L**) Representative confocal fluorescence images showing no apparent reporter (PVD neuron-marking GFP) alteration in *ergu-1* null mutants. (**M**) Representative confocal fluorescence images showing no apparent reporter (neuronal nuclear GCaMP6) alteration in *ergu-1* null mutants. (**N**) Representative confocal fluorescence images showing no apparent reporter (secreted *manf-1*::GFP) alteration by *ergu-1* RNAi. *P* value was determined by an unpaired *t*-test, two-tailed (comparison between two groups). [(A) to (K)] Scale bars, 100 μm. [(L) to (N)] Scale bars, 20 μm.
**Fig. S4. Importance of key cysteine residues for ERGU-1 functions.** (**A**) Representative fluorescence and bright-field images showing gst-4p::GFP expression in wild-type (WT), ergu-1 null mutants, and ergu-1 null mutants carrying transgenic extrachromosomal arrays expressing either wild-type or cysteine-mutant ergu-1 constructs. The *unc-54p*::mCherry co-injection marker labels body wall muscles. The C108A/C110A and C371A/C372A cysteine mutants failed to rescue the elevated gst-4p::GFP signal in the ergu-1 null background, indicating that both cysteine pairs are critical for ERGU-1 function. Right: Quantification of gst-4p::GFP fluorescence intensity. (**B**) Schematic of key residues for heme binding and interactions. Heme binding pocket is in high confidence region (90 > plDDT > 70). (**C**) Representative fluorescence and bright-field images showing gst-4p::GFP expression in wild-type (WT), ergu-1 null mutants, and ergu-1 null mutants carrying transgenic extrachromosomal arrays expressing either wild-type or tyrosine-mutant ergu-1 constructs. The *myo-2p*::mCherry co-injection marker labels pharynx. The Y454 tyrosine mutants failed to rescue the elevated gst-4p::GFP signal in the ergu-1 null background. Right: Quantification of gst-4p::GFP fluorescence intensity. (D) Multiple sequence alignment of *ergu-1* homologs from *C. elegans* (ce), *D. melanogaster* (dm), *D. rerio* (dr), *M. musculus* (mm), and *H. sapiens* (hs), highlighting conserved cysteine and tyrosine residues. Cysteine residues corresponding to C108, C110, C371, and C372, along with Y454, are boxed in red dashed lines. (E) AlphaFold2-predicted structures of ERGU-1 orthologs from the same five species. Conserved cysteine residues are shown as spheres in distinct colors for visualization. ****P < 0.0001; ns, not significant. [(A) and (C)] Scale bars, 100 μm.
**Fig. S5. Colocalization of ERGU-1::GFP with RFP-marked ER organelle reporters but not non-ER organelle reporters. (A)** Representative confocal fluorescence images showing colocalization between ERGU-1::GFP (green) and endoplasmic reticulum-specific reporters (red). Scale bars, 10 μm. (**B**) Representative confocal fluorescence images showing no colocalization between ERGU-1::GFP (green) and lysosome, mitochondria or plasma membrane-specific RFP reporters (red). Arrow indicates the organelles. Scale bars, 5 μm.
**Fig. S6. Protein characteristics of human TMEM161B, an ERGU-1 ortholog and evolutionarily conserved member of the ERGU protein family.** (**A**) Peak fractions (12.5) of human TMEM161B-2X Strep-HA protein purified by SEC in a non-reductive environment. (**B**) Peak fractions (14, 16) of TMEM161B-2X Strep-HA protein purified by SEC in a DTT-containing reductive environment. (**C**) TMEM161B-GFP in a non-reductive environment shown by Cryo-EM. (**D**) Peak of TMEM161B-GFP in a non-reductive environment shown by SEC. (**E**) Peak fractions (11.7) of TMEM161B-GFP protein purified by SEC in a non-reductive environment. (**F**) Immunofluorescence images showing that Human TMEM161A/B localizes to the ER membrane in U2OS cells. Scale bars, 10 μm. (**G**) Representative confocal fluorescence images of roGFP2-Orp1 detecting cytosolic H_2_O_2_ in 293T cells overexpressing human TMEM161A or B. Scale bars, 20 μm.
**Fig. S7: TMEM161B interacts with TMX2 and regulates ER stress in *C. elegans*.** (**A**) Predicted binding partners of human TMEM161B based on the human protein–protein interaction database. TMX2 ranks as the top predicted interactor with the highest confidence scores from two independent models. (**B**) Predicted structural model of the TMEM161B–TMX2 complex, illustrating the potential interface between the transmembrane helices of TMEM161B (green) and TMX2 (blue). (**C**) Representative epifluorescence images showing activation of the *hsp-4*p::GFP ER stress reporter following *ergu-1* and *C35D10.10* RNAi treatment. Quantification (right) shows that both knockdowns induce comparable *hsp-4*p::GFP fluorescence levels. Scale bars, 100 μm.
**Table S1: ERGU-1–associated proteins identified by co-immunoprecipitation and mass spectrometry.** Representative metabolic proteins identified from GFP-Trap co-immunoprecipitation of *ergu-1*::GFP compared with *ergu-1* RNAi worms are shown.

Data S1. Custom Python scripts utilized in UCSF ChimeraX to execute the AlphaFold2-assisted computational structural screening and distance measurements of cysteine pairs, related to Figure 1A and Figure S1

SUPPLEMENTAL INFORMATION

Supplemental information can be found online at https://doi.org/10.1016/j.celrep.2026.117489.

## Figures and Tables

**Figure 1.**
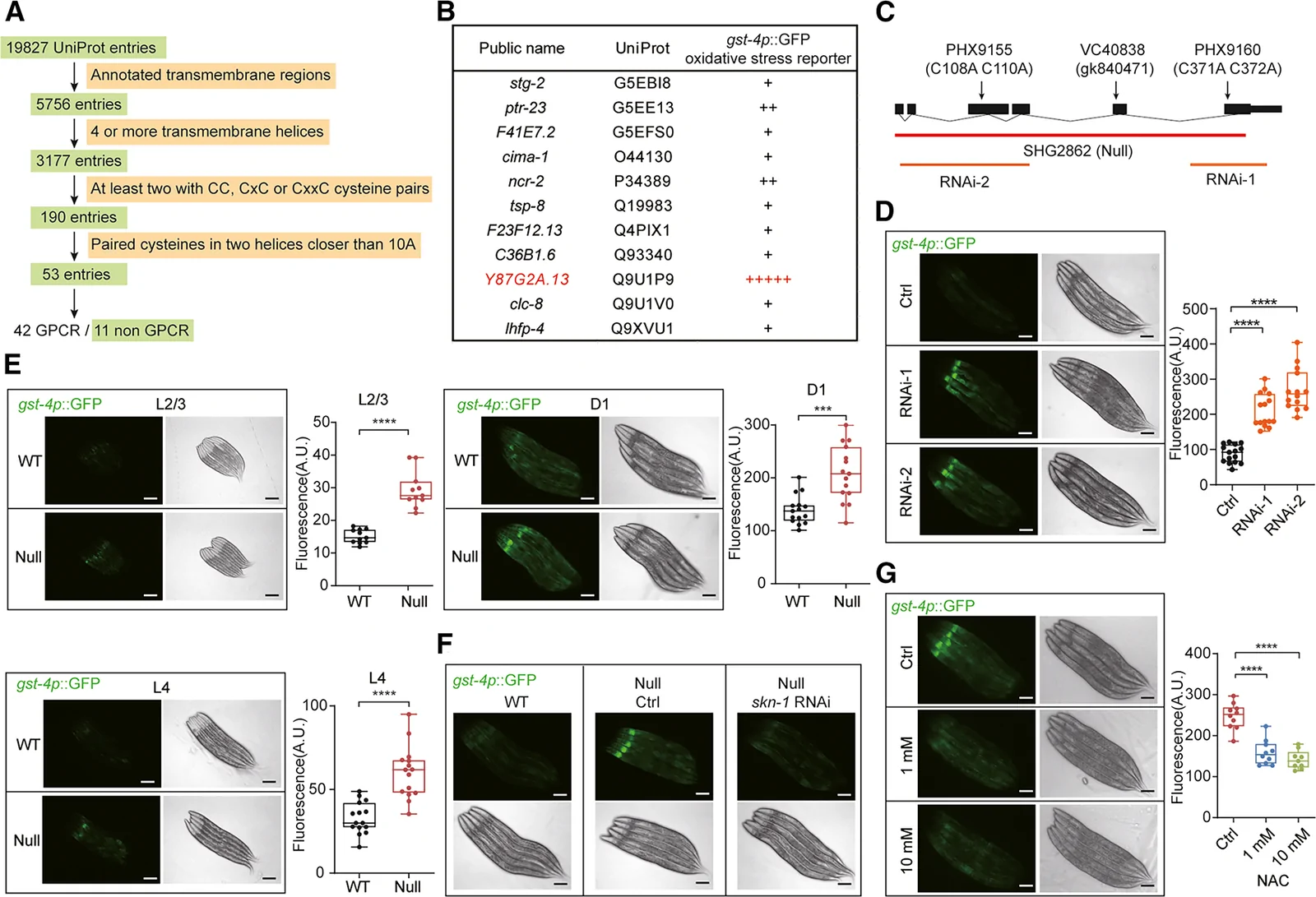
Computational and genetic identification of ERGU-1 (A) Schematic of AlphaFold2-assisted computational screens. (B) Phenotype-driven functional screens based on RNAi activation of *gst-4p::GFP* oxidative stress reporters. (C) Gene structure of *ergu-1* showing various alleles. (D and E) Representative epifluorescence images showing *gst-4p::GFP* activation in two independent *ergu-1* RNAi (D) and mutants (E) (*n* = 15 for each group). (F and G) Representative epifluorescence images showing that *gst-4p::GFP* activation in *ergu-1* null mutants requires ROS and *skn-1* (F), which are suppressed by NAC dose-dependently (G) (*n* = 10 for each group), with quantification of *gst-4p*:GFP fluorescence intensities under conditions indicated. *p* value was determined by an unpaired *t* test, two-tailed (comparison between two groups) or one-way ANOVA (comparison between multiple groups). Scale bars, 100 μm. Data are represented as mean ± SEM.

**Figure 2.**
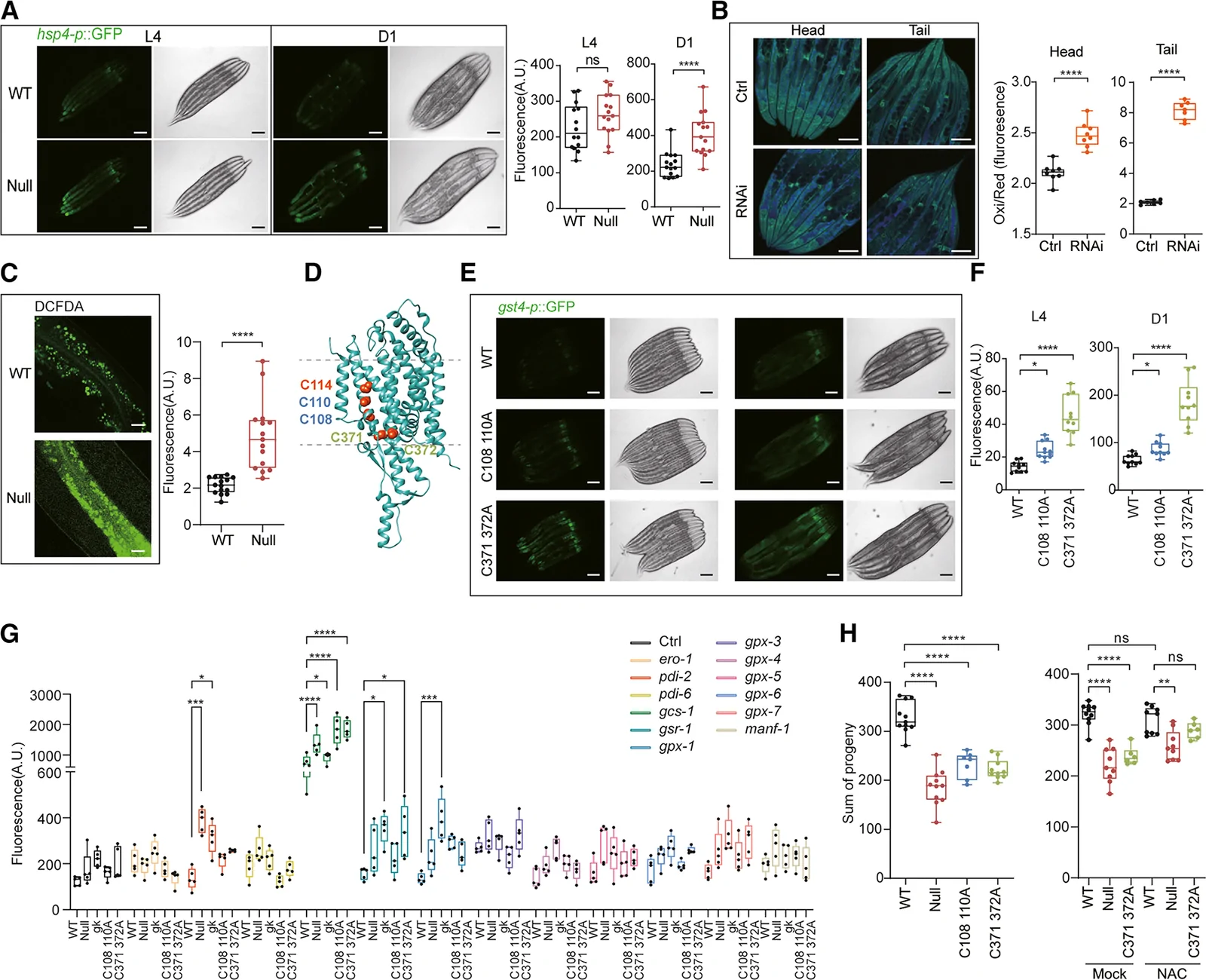
ERGU-1 defends against H_2_O_2_ and maintains organismal functions (A) Representative epifluorescence images showing increased *hsp-4p::GFP* in *ergu-1* null mutants (*n* = 15 for each group). (B) Representative confocal images showing increased oxidation/reduction ratio of Orp1-roGFP (H_2_O_2_ indicator) with *ergu-1* RNAi. (C) DCFDA staining showing increased DCF in null mutants (*n* = 15 for each group). (D) AlphaFold2-predicted structure (AF-Q9U1P9-F1-v4) revealing cysteine residues that likely mediate electron transfer across membranes. Cysteine residues are highlighted in orange spheres, and transmembrane segments are indicated by dotted lines. (E) Representative epifluorescence images showing increased *gst-4p*::*GFP* in two different cysteine pair mutants. (F) Quantification of *gst-4p*::*GFP* fluorescence intensities in WT and two different cysteine pair mutants indicated (*n* = 10 for each group). (G) Fluorescence intensity of *gst-4p*::*GFP* in WT and *ergu-1* null mutants under control (Ctrl) and RNAi conditions targeting redox regulators, including *ero-1*, *pdi-2*, *pdi-6*, *gcs-1*, *gsr-1*, *gpx-1*, *gpx-3*, *gpx-4*, *gpx-5*, *gpx-6*, *gpx-7*, and *manf-1*. (H) Quantification of brood sizes in WT, *ergu-1* null and cysteine mutants (left). Comparison of brood sizes between WT and mutant strains under mock and 10 mM NAC treatments (right). *p* value was determined by an unpaired *t* test, two-tailed (comparison between two groups) or one-way ANOVA (comparison between multiple groups). Data are represented as mean ± SEM. [(A), (B), and (E)]. Scale bars, 100 μm. (C) Scale bars, 20 μm.

**Figure 3.**
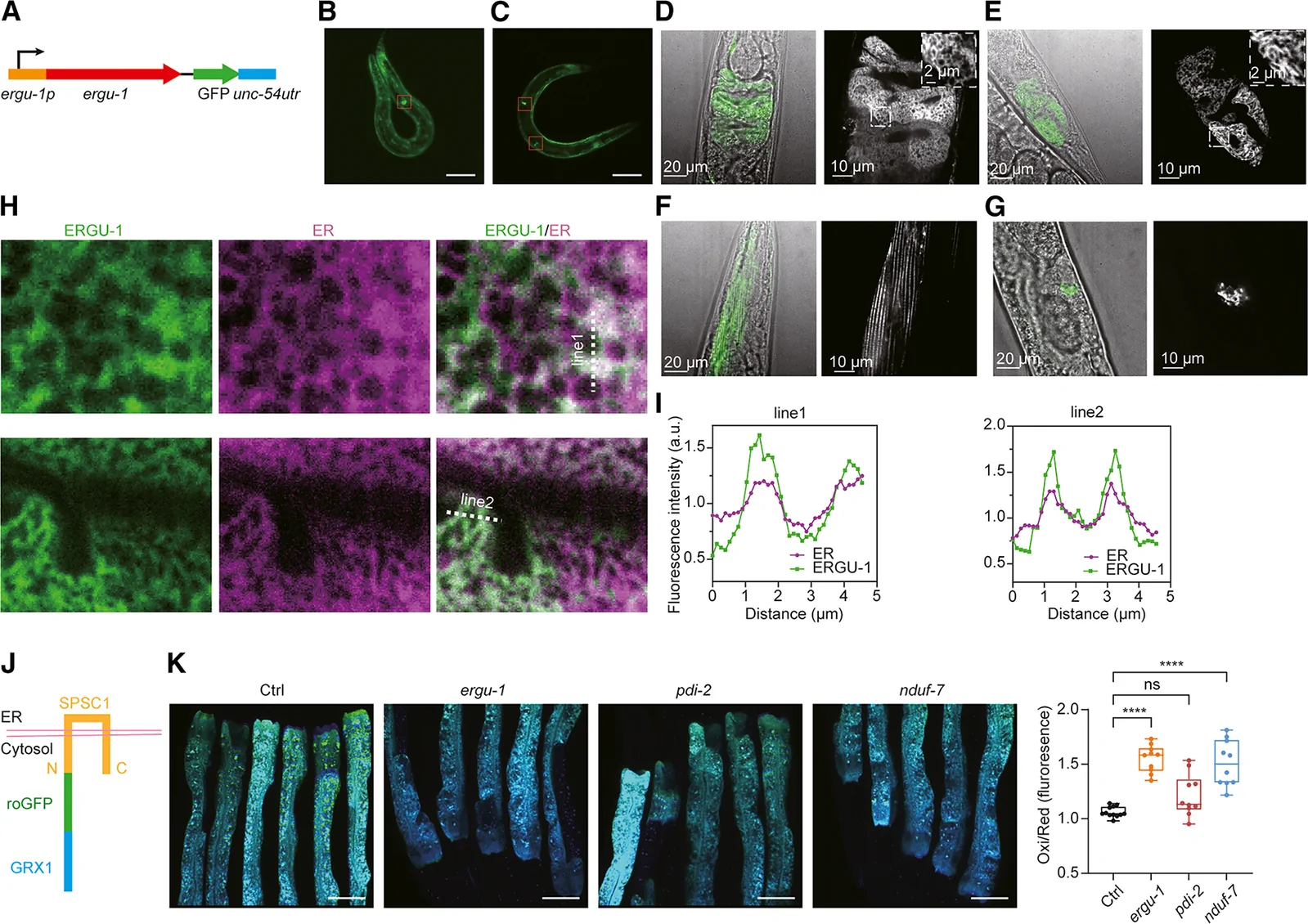
ERGU-1 forms a reticular network at the ER membrane (A) Schematic of the *ergu-1*p::*ERGU-1::GFP* translation reporter. (B and C) Representative low-mag view of *ergu-1p::GFP* (B) and *ergu-1p*::*ERGU-1::GFP* (C), respectively, showing expression in anterior and posterior intestine, body wall muscles and spermatheca. Scale bars, 100 μm. (D–G) Representative high-mag confocal view of *ergu-1*p:*ERGU-1::*GFP showing protein localization and expression in anterior (D) and posterior (E) intestinal cells, body wall muscles (F) and spermatheca (G). (H) Representative high-mag confocal view of ERGU-1GFP proteins showing co-localization with the ER membrane marker SEL-1(1–79)mCherryHDEL in the intestine, body wall muscles, and spermatheca. Scale bars, 100 μm. (I) Line scans showing co-localization. (J) Schematic design of the ER-proximal H_2_O_2_ sensor GRX1-roGFP-SPSC-1. (K) Representative ratiometric images and quantification of elevated H_2_O_2_ levels in the cytosol near the ER under *ergu-1* and *nduf-7* RNAi treatment, as indicated by the ER-proximal H_2_O_2_ sensor. Scale bars, 100 μm. Data are represented as mean ± SEM.

**Figure 4.**
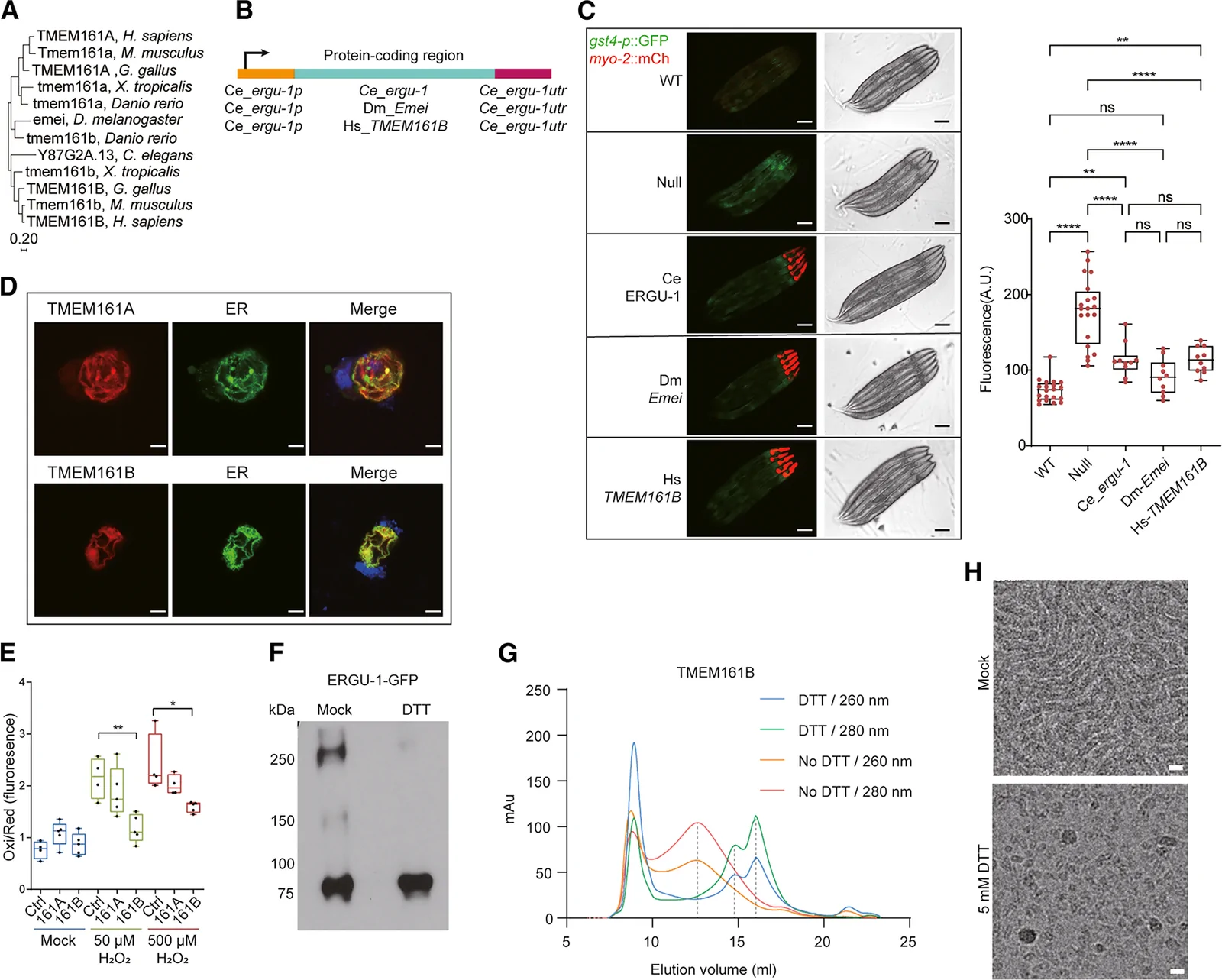
Conserved ER localization and antioxidant roles of ERGU-1 homologs (A) Maximum likelihood-based phylogenetic tree of ERGU-1 family proteins. (B) Schematic representation of transgenic constructs and strategy to rescue *ergu-1* null phenotype. (C) Representative fluorescence images of *gst-4p::GFP* showing rescue of *C. elegans ergu-1* mutants by *Drosophila* and human homologs (*n* = 10 for each rescue group). Quantification (right). Scale bars, 100 μm. (D) Immunofluorescence images showing that human TMEM161A and TMEM161B (tagged with mCherry) localize to the ER membrane, as indicated by co-localization with an ER-targeted GFP marker in HEK293 cells. Nuclei are counterstained with DAPI (blue). Scale bars, 10 μm. (E) Reduction of cytosolic H_2_O_2_ detected by roGFP2-Orp1 in 293T cells overexpressing human TMEM161A or TMEM161B. (F) Representative SDS-PAGE western blots with antibodies against GFP showing formation of oligomers (likely dimers and tetramers based on the molecular weight) by ERGU-1GFP and reduction to monomers by 10 mM dithiothreitol (DTT). (G) Chromatography plot showing reduction of purified human TMEM161B to smaller size peaks in the presence of 5 mM DTT as analyzed by SEC. (H) Reduction of TMEM161B strep-HA from filamentary forms to monomers or dimers in the presence of 5 mM DTT as shown by cryo-electron microscopy. Scale bars, 10 nm. (C and E) *p* value was determined by one-way ANOVA. Data are represented as mean ± SEM.

**KEY RESOURCES TABLE:** 

REAGENT or RESOURCE	SOURCE	IDENTIFIER

Bacterial and virus strains		

*Escherichia coli* HT115(DE3)	*Caenorhabditis* Genetics Center	N/A
*Escherichia coli* C3040H	NEB	N/A
*Escherichia coli* OP50	*Caenorhabditis* Genetics Center	Wormbase ID: OP50
Ahringer RNAi library	Source BioScience	N/A

Chemicals, peptides, and recombinant proteins		

5-fluoro-2-deoxyuridine	Sigma-Aldrich (St Louis, MO, USA)	50-91-9
Sodium hydroxide	Fisher Scientific (Pittsburg, PA, USA)	1310-73-2
Sodium hypochlorite	Fisher Scientific (Pittsburg, PA, USA)	7681-52-9
Isopropyl-beta-D-thiogalactoside	Fisher Scientific (Pittsburg, PA, USA)	367-93-1
Ampicillin	Sigma-Aldrich (St Louis, MO, USA)	69-53-4
Methyl *tert*-butyl ether	Sigma-Aldrich (St Louis, MO, USA)	1634-04-4
Isopropanol	Sigma-Aldrich (St Louis, MO, USA)	67-63-0
Ammonium acetate	Sigma-Aldrich (St Louis, MO, USA)	631-61-8
FerroOrange	Dojindo (ROCKVILLE, MD, USA)	F374-12
CM-H2DCFDA (General Oxidative Stress Indicator)	Invitrogen	C6827
MitoTracker^®^ red CM-H2XRos	Fisher Scientific (Pittsburg, PA, USA)	M46752

Deposited data		

https://doi.org/10.5281/zenodo.19673017	original code at Zenodo	
https://data.mendeley.com/datasets/fgg6sft85z/1	Mendeley data	

Experimental models: Organisms/strains		

N2 Bristol strain (wild type)	CGC	N2
*ergu-1(ust572)*	Guang lab	SHG2862
*ergu-1(C108/110A)*	SunyBiotech	PHX9153
*ergu-1(C371/372A)*	SunyBiotech	PHX9160
*ergu-1(gk840471)*	CGC	VC40838
*gst-4p::GFP*	CGC	CL2166
*hsp-4p::GFP*	CGC	SJ4005
*ergu-1p:: ergu-1::gfp*	Ma lab	DMS2524
*ergu-1p::hsTMEM161B::ergu-1utr; myo-2p::mCherry*	Ma lab	DMS2701
*ergu-1p::ergu-1::ergu-1utr; myo-2p::mCherry*	Ma lab	DMS2702
*ergu-1p::Dm-161:: ergu-1utr; myo-2p::mCherry*	Ma lab	DMS2704
*rps-0p::roGFP2-Orp1*	Bart Braeckman Lab	JV10
*jrIs2 [rpl-17p::Grx1-roGFP2 + unc-119(+)]*	CGC	JV2
*ergu-1 (gk840471) I; jrIs2*	Ma lab	DMS2564
*hrg-1p::gfp*	Hamza Lab	IQ6011
*pwIs890[Pvha-6::AKT(PH)::GFP]*	Ma lab	DMS2555
*pwIs503 [vha-6p::mans::GFP + Cbr-unc-119(+)]*	CGC	RT1315
*vit-2(crg9070[vit-2::gfp]) X*	CGC	BCN9071
*vkIs2877 [nhx-2p::sqst-1::CemOrange2 + myo-2p::GFP]*	CGC	VK2877
*wwIs24 [acdh-1p::GFP + unc-119(+)]*	Walhout Lab	VL749
*xbIs1502 [act-1::GFP + rol-6(su1006)]*	CGC	UN1502
*wdIs52 [F49H12.4::GFP + unc-119(+)]*	CGC	NC1687
*dmaIs11[Pmanf-1::manf-1 ::GFP*	Ma lab	DMS179
*dvIs19 III; tmem-161(ust572)*	Ma lab	DMS2639
*zcIs4 V; tmem-161(ust572)*	Ma lab	DMS2644
*hpIs675[rgef-1p::GCaMP6s::3xNLS::mNeptune + lin-15(+)]; tmem-161(ust572)*	Ma lab	DMS2652
*dvIs19 III; tmem-161(ust572)*	Ma lab	DMS2661
*tmem-161(ust572); dmaIs11*	Ma lab	DMS2676
*tmem-161(ust572); pwIs98*	Ma lab	DMS2677
*otIs77 II; wyIs592; tmem-161(ust572)*	Ma lab	DMS2670
*kaIs12; tmem-161(ust572)*	Ma lab	DMS2671
*tmem-161(ust572); dmaIs160*	Ma lab	DMS2942
*hpIs675 [rgef-1p::GCaMP6s::3xNLS::mNeptune + lin-15(+)]*	CGC	ZM9624
*ihIs35 [yap-1::GFP::unc-54 3′UTR + lin-15(+)]; tmem-161(ust572)*	Ma lab	DMS2672

Oligonucleotides		

*ergu-1* RNAi 1	GGCCCCCCCTCGAGGTCGACAGGAGACGATGCTATAAACTATGCT	TCCACCGCGGTGGCGGCCGCCGTGACGTGTTTTCAGCTCG
*ergu-1* RNAi 2	GGCCCCCCCTCGAGGTCGACATGCCAAAATGAATGCGGGC	TCCACCGCGGTGGCGGCCGCACGTGGTGATATGCCTGGTG

Software and algorithms		

PRISM v10	GraphPad Software	https://www.graphpad.com/updates/prism-10-2-0-release-notes
ImageJ	NIH	https://imagej.net/ij/index.html
WormLab System	MBF Bioscience	https://www.mbfbioscience.com/products/wormlab-imaging-system
Jalview	Jalview	https://www.jalview.org/
ChimeraX	UCSF RBVI	https://www.cgl.ucsf.edu/chimerax/

## Highlights

- AlphaFold screen identifies ERGU-1 as a conserved regulator of ER redox homeostasis
- ERGU-1 buffers ER-derived H_2_O_2_ to maintain proteostasis and animal reproduction
- Human TMEM161B and *Drosophila* homologs rescue *C. elegans* ERGU-1 mutants
- ERGU-1 and human TMEM161B assemble into redox-modulated oligomeric structures
