# Supplementary material for: A conserved antioxidant defense at the endoplasmic reticulum membrane
Source: Cell Rep. Author manuscript; Available in PMC 2026 Oct 27. (PMC13713018; doi:10.1016/j.celrep.2026.117489)
Supplement: Supplementary file 1 [file NIHMS2212390-supplement-Document_S1__Figures_S1_S7_and_Table_S1.pdf]

Cell Reports, Volume *45*

Supplemental information

A conserved antioxidant defense

at the endoplasmic reticulum membrane

Zhijian Ji, Henry de Belly, Taruna Pandey, Bingying Wang, Yao Tang, Jingxuan Yao, Shiya Xu, Kathy Li, Yanchang Bian, Shouhong Guang, Zhiyong Lou, Al Burlingame, Orion D. Weiner, Thomas D. Goddard, and Dengke K. Ma

A

Organism	Blast Name	Score	Number of Hits	Description
Eukaryota	eukaryotes		10	
·Sar	eukaryotes		2	
··Reticulomyxa filosa	forams	248	1	Reticulomyxa filosa hits
··Symbiodinium pilosum	dinoflagellates	95.1	1	Symbiodinium pilosum hits
·Acer saccharum	eudicots	224	1	Acer saccharum hits
·Ipomoea batatas	eudicots	151	1	Ipomoea batatas hits
·Culicoides impunctatus	flies	93.6	1	Culicoides impunctatus hits
·Idotea baltica	isopods	87	2	Idotea baltica hits
·Friedmanniomyces endolithicus	ascomycete fungi	68.6	3	Friedmanniomyces endolithicus hits

B

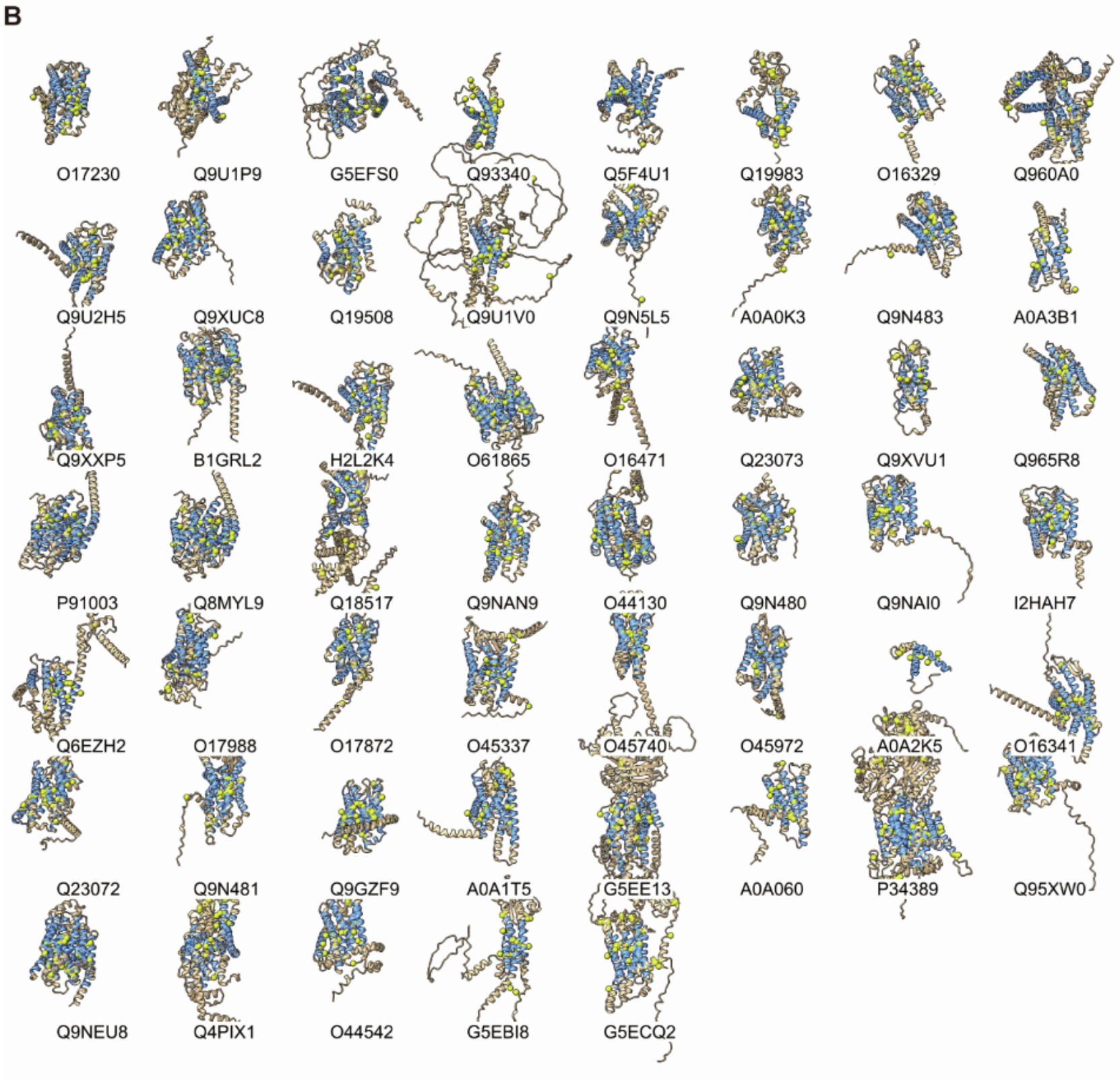

C

Gene Name	Automated Description
stg-2	Predicted to enable channel regulator activity and voltage-gated calcium channel activity. Involved in adult locomotory behavior and positive regulation of glutamatergic synaptic transmission. Predicted to be located in postsynaptic density membrane. Predicted to be part of AMPA glutamate receptor complex.
ptr-23	Involved in endocytosis and molting cycle. Acts upstream of or within nematode male tail mating organ morphogenesis. Predicted to be located in cytoplasmic vesicle membrane and plasma membrane. Expressed in hypodermis.
F41E7.2	Predicted to enable antiporter activity. Predicted to be involved in proton transmembrane transport. Predicted to be located in membrane. Is an ortholog of human SLC9B1 (solute carrier family 9 member B1) and SLC9B2 (solute carrier family 9 member B2).
cima-1	Predicted to enable sialic acid transmembrane transporter activity. Involved in regulation of protein catabolic process and regulation of synapse organization. Located in lysosome. Expressed in hypodermis and intestine.
ncr-2	Predicted to enable cholesterol binding activity. Involved in cholesterol homeostasis\; cholesterol transport\; and nematode larval development. Predicted to be located in plasma membrane. Expressed in XXXL\; XXXR\; gonadal sheath cell\; spermatheca\; and ventral nerve cord. Used to study Niemann-Pick disease. Human ortholog(s) of this gene implicated in Niemann-Pick disease type C1. Is an ortholog of human NPC1 (NPC intracellular cholesterol transporter 1).
tsp-8	Predicted to be located in membrane.
F23F12.13	Predicted to enable transmembrane transporter activity. Predicted to be involved in transmembrane transport. Predicted to be located in membrane. Is an ortholog of human SLC22A15 (solute carrier family 22 member 15) and SVOPL (SVOP like).
C36B1.6	Predicted to be located in membrane.
Y87G2A.13	Predicted to be located in membrane. Is an ortholog of human TMEM161A (transmembrane protein 161A).
clc-8	Predicted to be located in membrane.
lhfp-4	Predicted to be involved in sensory perception of sound. Predicted to be located in plasma membrane. Human ortholog(s) of this gene implicated in autosomal recessive nonsyndromic deafness 67. Is an ortholog of human LHFPL4 (LHFPL tetraspan subfamily member 4).

Fig. S1. AlphaFold2-based computational screen for ERGU candidates. (**A**) BLASTP search yielding no apparent broadly conserved eukaryotic homologs of DsbD/ScsB based on protein sequence similarities, prompting a search for DsbD/ScsB homologs based on functional and structural features using AlphaFold2-predicted structures. (**B**) Predicted structures of the 53 ERGU candidates identified. Transmembrane helices are shown in blue, cysteine residues in yellow, and the remainder of the protein in gold. (**C**) Table listing the annotated functions of the 11 non-GPCR ERGU candidates.

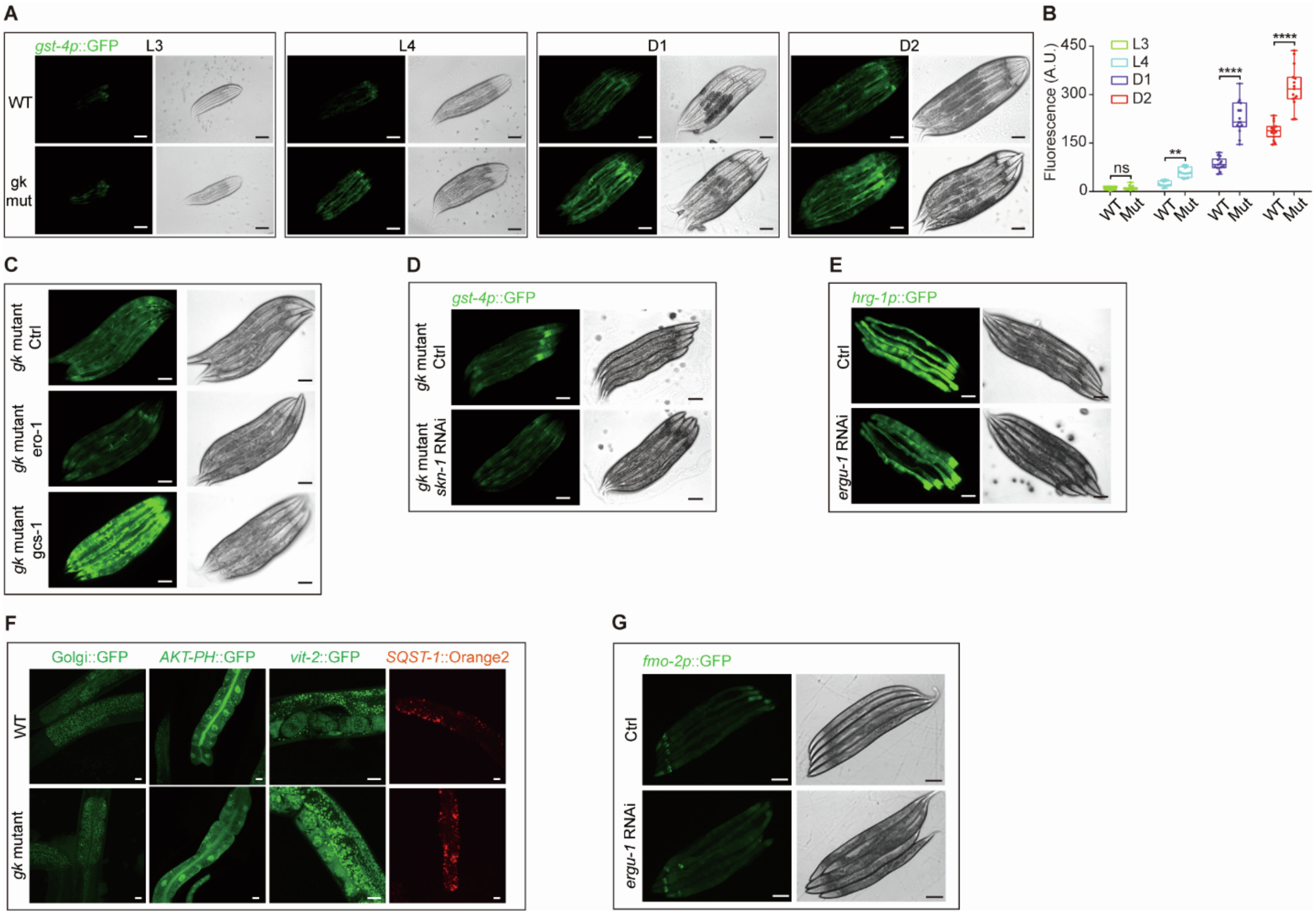

Fig. S2. Phenotypes of ERGU-1 protein-truncating *ergu-1(gk840471)* mutants. (**A**) Representative epifluorescence images showing *gst-4p::GFP* activation in *ergu-1*(*gk840471*) mutants (n=15 for each group). (**B**) Quantification of *gst-4p*::GFP fluorescence intensities in WT and *ergu-1*(*gk840471*) mutants indicated (n=15 for each group). (**C**) Representative epifluorescence images showing *gst-4p::*GFP decrease by *ero-1* RNAi and increase by *gcs-1* RNAi (**D**) Representative epifluorescence images showing *gst-4p::GFP* activation in *ergu-1*(*gk840471*) mutants requires *skn-1.* (**E**) Representative fluorescence and corresponding bright-field images showing *hrg-1p*::GFP reporter expression in control and ergu-1 RNAi-treated worms. (**F**) Representative confocal fluorescence images showing no apparent reporter (Golgi

apparatus-marking Golgi::GFP, apical and endosomal membrane-marking *akt-ph*::GFP, yolk organelle-marking *vit-2*::GFP and autophagosome-marking *sqst-1*::mOrange2) alteration in *ergu-1*(*gk840471*) mutants. (**G**) Representative fluorescence and corresponding bright-field images showing *fmo-2p*::GFP reporter expression in control and ergu-1 RNAi-treated worms. *P* value was determined by an unpaired *t*-test, two-tailed (comparison between two groups). [(A), (B), (C), (E) and (G)] Scale bars, 100 μm. (D) Scale bars, 20 μm.

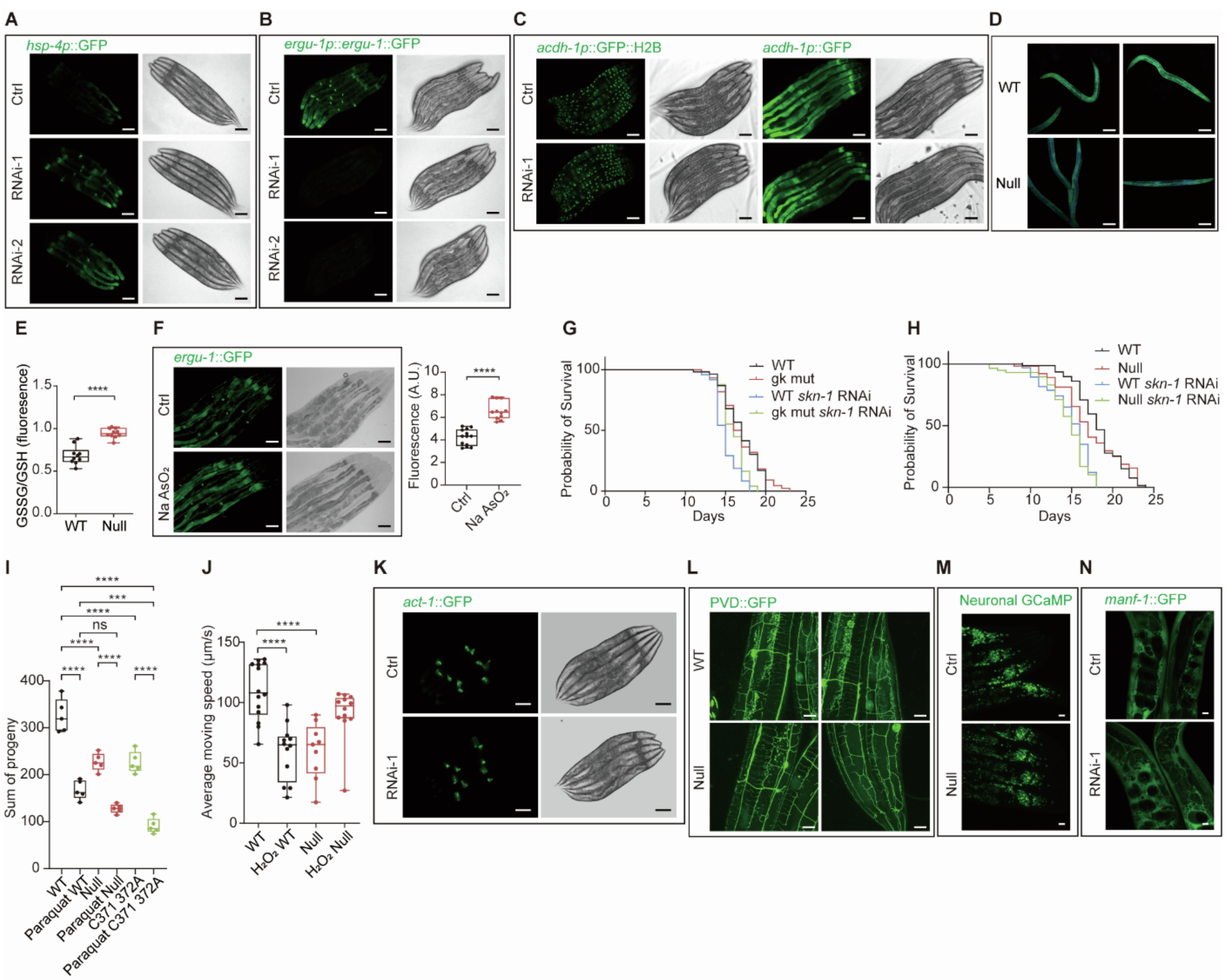

Fig. S3. Phenotypic consequences of RNAi or mutant *ergu-1*. (**A**) Representative epifluorescence images showing increased *hsp-4p::GFP* upon *ergu-1* RNAi treatment.

(**B**) Representative fluorescence images of *ergu-1p*::*ergu-1::GFP* showing abolished GFP signals by *ergu-1* RNAi. (**C**) Representative fluorescence images of Vitamin B12 stress reporter *acdh-1p*:: *GFP* showing unaltered GFP signals by *ergu-1* RNAi. (**D** and **E**) Representative fluorescence images of glutathione stress GSSG/GSH reporters showing elevated oxidative stress in *ergu-1* null mutants (D) and quantification (n=10) (E). (**F**) Representative fluorescence images of *ergu-1p*::*ergu-1::GFP* showing reporter up-regulation by oxidative stressors (0.5% $NaAsO_2$). (**G**) Lifespan curves of wild-type and *ergu-1* null mutants with control or *skn-1* RNAi showing comparable lifespans in wild-type and *ergu-1* null mutants and reduction by *skn-1* RNAi. (**H**) Lifespan curves of wild-type and *ergu-1* protein-truncating mutants (*gk840471*) with control or *skn-1* RNAi comparable lifespans in wild-type and *ergu-1(gk840471)* mutants and reduction by *skn-1* RNAi ($n \geqslant 50$). (**I**) Quantification of brood sizes in wild type, *ergu-1* null and cysteine mutants upon 10 mM paraquat treatment. *P* value was determined by one-way ANOVA (comparison between multiple groups). (**J**) Quantification of average moving speed in wild-type, ergu-1 null and above strain treated with 10 mM H2O2. (**K**) Representative fluorescence images of cytoskeletal actin *act-1*::GFP reporters showing no apparent alteration by *ergu-1* RNAi. (**L**) Representative confocal fluorescence images showing no apparent reporter (PVD neuron-marking GFP) alteration in *ergu-1* null mutants. (**M**) Representative confocal fluorescence images showing no apparent reporter (neuronal nuclear GCaMP6) alteration in *ergu-1* null mutants. (**N**) Representative confocal fluorescence images showing no apparent reporter (secreted *manf-1*::GFP) alteration by *ergu-1* RNAi. *P* value was determined by an unpaired *t*-test, two-tailed (comparison between two groups). [(A) to (K)] Scale bars, 100 μm. [(L) to (N)] Scale bars, 20 μm.

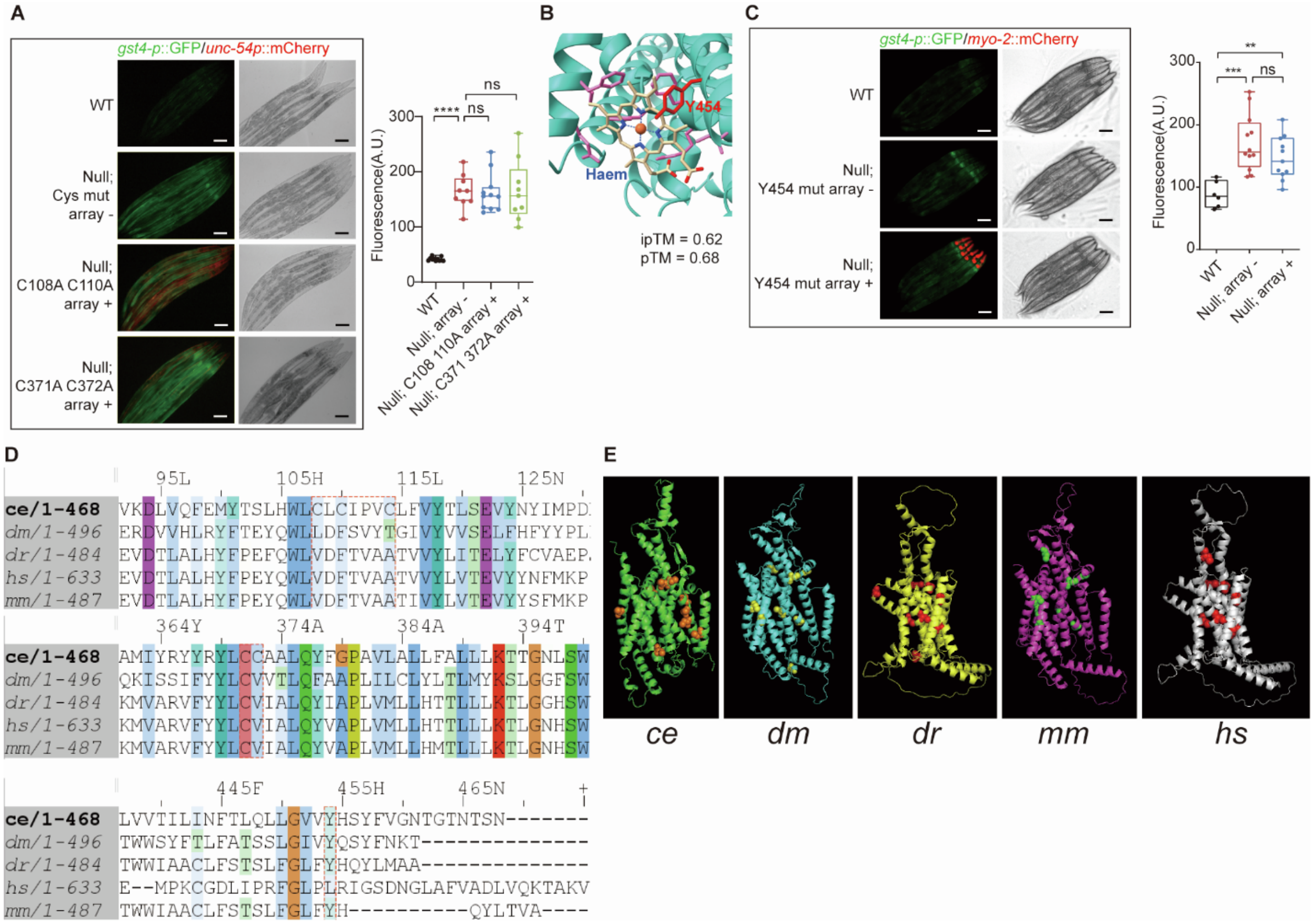

Fig. S4. Importance of key cysteine residues for ERGU-1 functions. (**A**) Representative fluorescence and bright-field images showing gst-4p::GFP expression in wild-type (WT), ergu-1 null mutants, and ergu-1 null mutants carrying transgenic extrachromosomal arrays expressing either wild-type or cysteine-mutant ergu-1 constructs. The *unc-54p*::mCherry co-injection marker labels body wall muscles. The C108A/C110A and C371A/C372A cysteine mutants failed to rescue the elevated gst-4p::GFP signal in the ergu-1 null background, indicating that both cysteine pairs are critical for ERGU-1 function. Right: Quantification of gst-4p::GFP fluorescence intensity. (**B**) Schematic of key residues for heme binding and interactions. Heme binding pocket is in high confidence region (90 > plDDT > 70). (**C**) Representative fluorescence and

bright-field images showing gst-4p::GFP expression in wild-type (WT), ergu-1 null mutants, and ergu-1 null mutants carrying transgenic extrachromosomal arrays expressing either wild-type or tyrosine-mutant ergu-1 constructs. The *myo-2p*::mCherry co-injection marker labels pharynx. The Y454 tyrosine mutants failed to rescue the elevated gst-4p::GFP signal in the ergu-1 null background. Right: Quantification of gst-4p::GFP fluorescence intensity. (D) Multiple sequence alignment of *ergu-1* homologs from *C. elegans* (ce), *D. melanogaster* (dm), *D. rerio* (dr), *M. musculus* (mm), and *H. sapiens* (hs), highlighting conserved cysteine and tyrosine residues. Cysteine residues corresponding to C108, C110, C371, and C372, along with Y454, are boxed in red dashed lines. (E) AlphaFold2-predicted structures of ERGU-1 orthologs from the same five species. Conserved cysteine residues are shown as spheres in distinct colors for visualization. ****P < 0.0001; ns, not significant. [(A) and (C)] Scale bars, 100 μm.

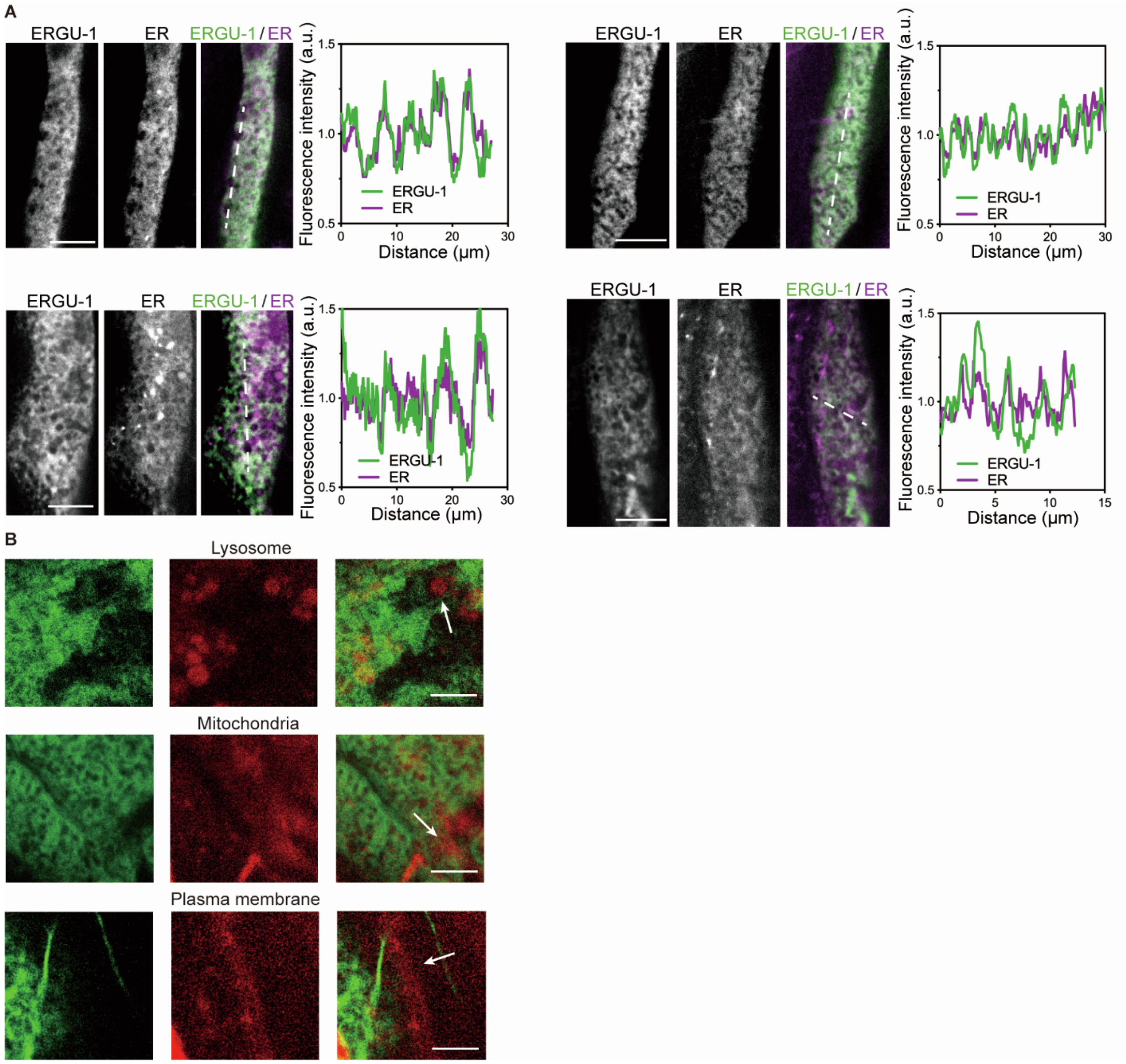

Fig. S5. Colocalization of ERGU-1::GFP with RFP-marked ER organelle reporters but not non-ER organelle reporters. (A) Representative confocal fluorescence images showing colocalization between ERGU-1::GFP (green) and endoplasmic reticulum-specific reporters (red). Scale bars, 10 μm. (**B**) Representative confocal fluorescence images showing no colocalization between ERGU-1::GFP (green) and lysosome, mitochondria or plasma membrane-specific RFP reporters (red). Arrow indicates the organelles. Scale bars, 5 μm.

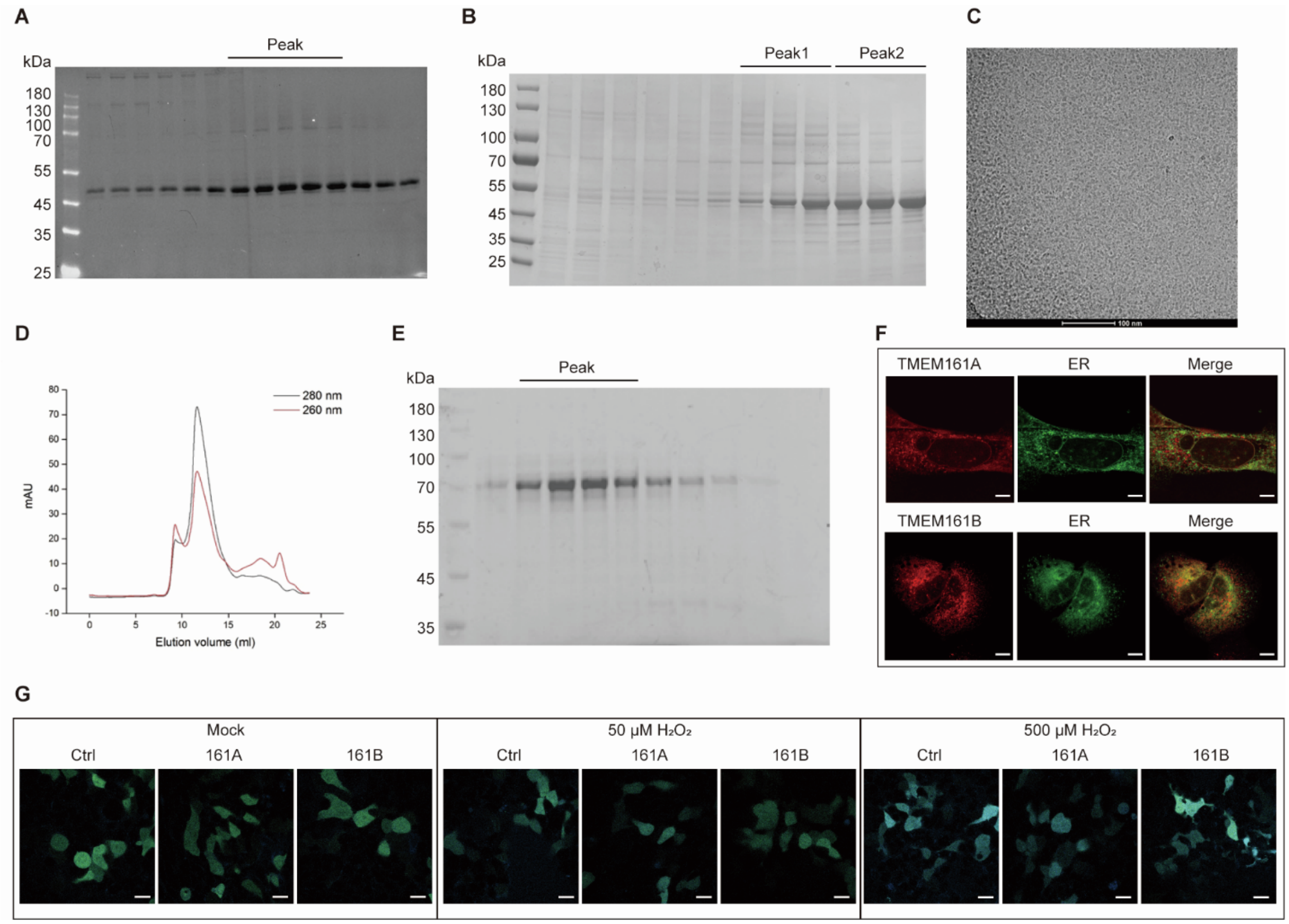

Fig. S6. Protein characteristics of human TMEM161B, an ERGU-1 ortholog and evolutionarily conserved member of the ERGU protein family. (**A**) Peak fractions (12.5) of human TMEM161B-2X Strep-HA protein purified by SEC in a non-reductive environment. (**B**) Peak fractions (14, 16) of TMEM161B-2X Strep-HA protein purified by SEC in a DTT-containing reductive environment. (**C**) TMEM161B-GFP in a non-reductive environment shown by Cryo-EM. (**D**) Peak of TMEM161B-GFP in a non-reductive environment shown by SEC. (**E**) Peak fractions (11.7) of TMEM161B-GFP protein purified by SEC in a non-reductive environment. (**F**) Immunofluorescence images showing that Human TMEM161A/B localizes to the ER membrane in U2OS cells. Scale bars, 10 μm. (**G**) Representative confocal fluorescence images of roGFP2-

Orp1 detecting cytosolic H_2O_2 in 293T cells overexpressing human TMEM161A or B. Scale bars, 20 µm.

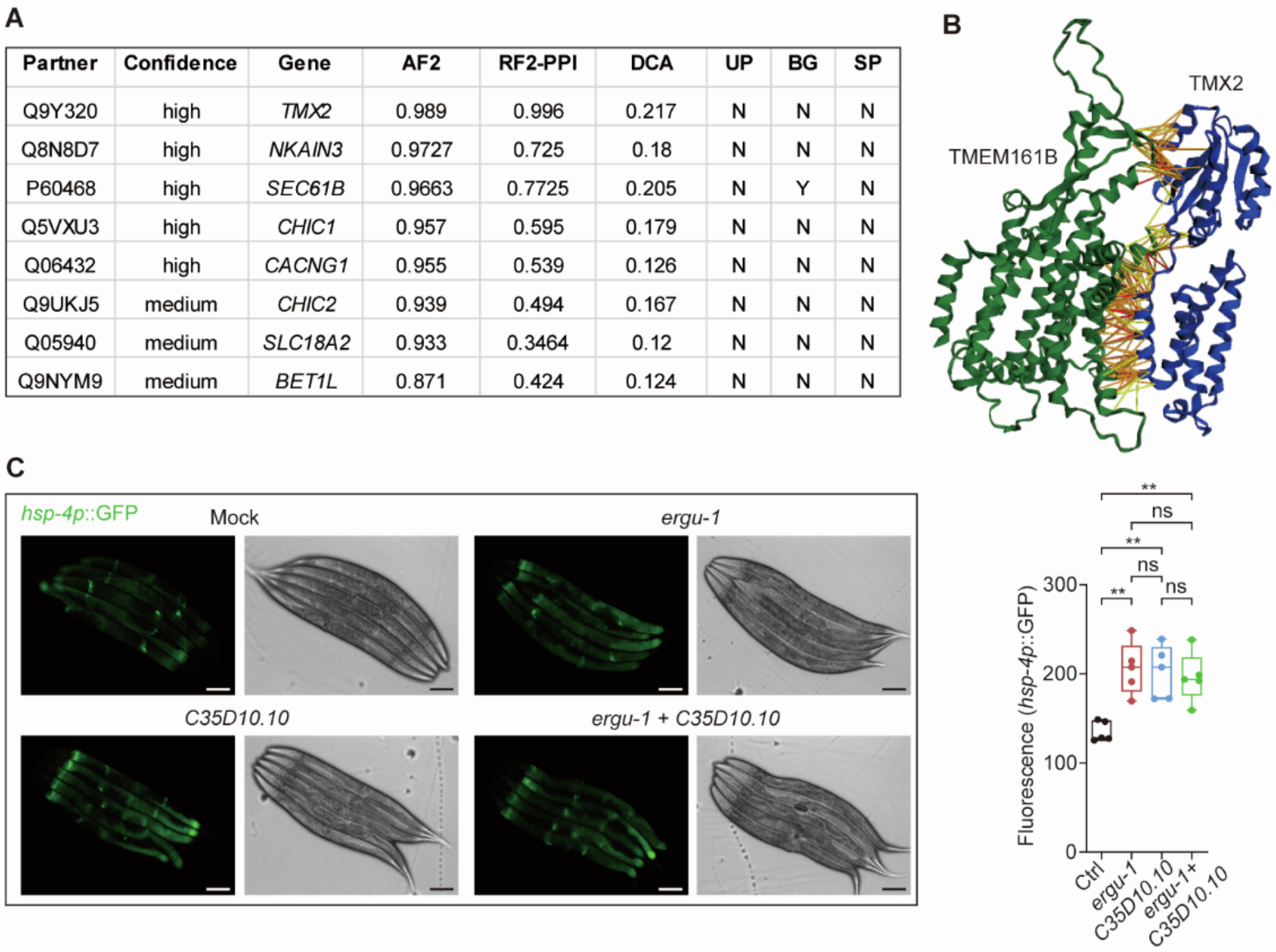

Partner	Confidence	Gene	AF2	RF2-PPI	DCA	UP	BG	SP
Q9Y320	high	*TMX2*	0.989	0.996	0.217	N	N	N
Q8N8D7	high	*NKAIN3*	0.9727	0.725	0.18	N	N	N
P60468	high	*SEC61B*	0.9663	0.7725	0.205	N	Y	N
Q5VXU3	high	*CHIC1*	0.957	0.595	0.179	N	N	N
Q06432	high	*CACNG1*	0.955	0.539	0.126	N	N	N
Q9UKJ5	medium	*CHIC2*	0.939	0.494	0.167	N	N	N
Q05940	medium	*SLC18A2*	0.933	0.3464	0.12	N	N	N
Q9NYM9	medium	*BET1L*	0.871	0.424	0.124	N	N	N

Fig. S7: TMEM161B interacts with TMX2 and regulates ER stress in *C. elegans*. (**A**) Predicted binding partners of human TMEM161B based on the human protein–protein interaction database. TMX2 ranks as the top predicted interactor with the highest confidence scores from two independent models. (**B**) Predicted structural model of the TMEM161B–TMX2 complex, illustrating the potential interface between the transmembrane helices of TMEM161B (green) and TMX2 (blue). (**C**) Representative epifluorescence images showing activation of the *hsp-4*p::GFP ER stress reporter following *ergu-1* and *C35D10.10* RNAi treatment. Quantification (right) shows that both knockdowns induce comparable *hsp-4*p::GFP fluorescence levels. Scale bars, 100 µm.

	ERGU-1::GFP ERGU-1 RNAi				ERGU-1::GFP Control				
Gene	Num Unique	Peptide Count	% Cov	Best Expect Val	Num Unique	Peptide Count	% Cov	Best Expect Val	Protein Name
CELE_Y87G2A.13					21	32	38.2	9.40E-19	Lysosomal cobalamin transporter
gdh-1	1	1	2.2	2.80E-10	15	15	31.9	2.80E-13	Glutamate dehydrogenase
gpd-2	3	3	11.7	8.00E-17	9	9	31.4	3.10E-15	Glyceraldehyde-3-phosphate dehydrogenase 2
act-1	5	9	23.9	3.30E-16	24	44	54.5	1.60E-19	Actin-1
rps-3	10	14	45.3	1.30E-14	28	44	80.2	3.50E-16	40S ribosomal protein S3
immt-1	1	1	2.1	1.20E-09	19	21	37.6	9.10E-15	MICOS complex subunit MIC60-1
asb-1					17	20	40.5	1.10E-15	ATP synthase F(0) complex subunit B1, mitochondrial
sec-61.a	1	1	2.7	1.40E-04	18	23	28.5	1.80E-14	Plug_translocon domain-containing protein
act-5	3	4	12.3	3.00E-10	18	33	55.7	5.10E-20	Actin
vdac-1	6	7	26.9	7.20E-14	21	33	79.2	1.50E-20	Probable voltage-dependent anion-selective channel
Y69A2AR.18	5	5	23.4	6.30E-13	20	39	49.5	2.00E-15	ATP synthase subunit gamma
haf-9	2	2	3.2	6.70E-09	16	17	25.9	7.60E-23	ABC type-1 domain-containing protein
mca-3	1	1	1.5	4.00E-09	15	15	15.8	4.30E-19	Calcium-transporting ATPase
atad-3	4	4	10.4	1.70E-11	18	19	32.1	1.70E-17	ATPase family AAA domain-containing protein 3
tbh-1	17	76	28.3	2.20E-20	30	42	38.7	1.50E-19	Tyramine beta-hydroxylase
eft-3	8	8	20.3	5.00E-13	21	23	45.6	4.50E-19	Elongation factor 1-alpha
rpl-4	5	12	23.8	4.50E-17	18	21	51.3	7.70E-19	60S ribosomal protein L4
atp-3	5	5	25.6	2.80E-12	18	32	65.7	1.00E-13	ATP synthase subunit O, mitochondrial
dim-1	3	3	5.9	2.80E-11	16	18	33.8	4.20E-16	Disorganized muscle protein 1

Table S1: ERGU-1–associated proteins identified by co-immunoprecipitation and mass spectrometry. Representative metabolic proteins identified from GFP-Trap co-immunoprecipitation of *ergu-1*::GFP compared with *ergu-1* RNAi worms are shown.
